# Estimating Methane Emission Durations Using Continuous Monitoring Systems

**DOI:** 10.1021/acs.estlett.4c00687

**Published:** 2024-10-28

**Authors:** William S. Daniels, Meng Jia, Dorit M. Hammerling

**Affiliations:** † Department of Applied Mathematics and Statistics, Colorado School of Mines, Golden, Colorado 80401, United States; ‡ Energy Emissions Modeling and Data Lab, The University of Texas at Austin, Austin, Texas 78712, United States

**Keywords:** methane, oil and gas, emission detection, emission duration, emission frequency, continuous monitoring systems, greenhouse gas reporting

## Abstract

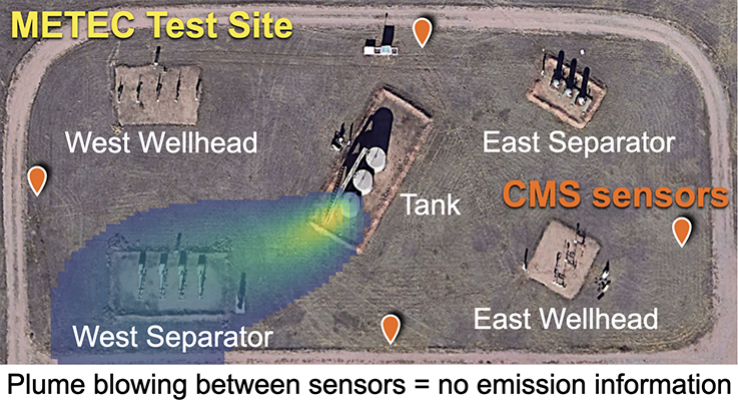

We propose a method for estimating methane emission durations on oil and gas sites, referred to as the Probabilistic Duration Model (PDM), that uses concentration data from continuous monitoring systems (CMS). The PDM probabilistically addresses a key limitation of CMS: nondetect times, or the times when wind blows emitted methane away from the CMS sensors (resulting in no detections). Output from the PDM can be used to bound the duration of emissions detected by survey-based technologies, such as plane or satellites, that have limited ability to characterize durations due to the typically low temporal frequency (e.g., quarterly) at which they observe a given source. Linear regression indicates that the PDM has a bias of −4.9% (R^2^ = 0.80) when evaluated on blinded controlled releases at the Methane Emissions Technology Evaluation Center (METEC), with 86.8% of estimates within a factor of 2× error from the true duration. We apply the PDM to a typical production site in the Appalachian Basin and use it to bound the duration of survey-based measurements. We find that failing to account for CMS nondetect times results in underestimated emission durations of up to a factor of 65× (6400%) on this site.

## Introduction

Updates to the EPA’s Greenhouse Gas Reporting Program (GHGRP) will require oil and gas operators to report maintenance or abnormal methane emissions greater than 100 kg/h starting in January 2025,^1^ including emissions identified by third parties (e.g., Carbon Mapper^2^). With more operators opting into voluntary aerial measurement campaigns and new methane-focused satellites (e.g., MethaneSAT^3^) providing publicly available data, the number of detected emissions meeting this reporting requirement is likely to increase.

A duration estimate is required for all emissions exceeding the 100 kg/h reporting threshold so that a total mass of methane can be computed and reported.^1^ Infrequent snapshot measurements from survey-based technologies have a limited ability to characterize emission durations due to the relatively low frequency at which they observe a given source. For example, an aerial measurement campaign measuring sites quarterly will only be able to bound emission start times at three month intervals, despite emissions potentially lasting for only a few hours or days.^4^ Satellites can provide more frequent measurements of a given source, but their current operational detection limits are greater than the 100 kg/h threshold, and cloud cover and surface albedo can also prevent detections.^5^

Higgins et al.^6^ propose methods for bounding emission durations using operational data (e.g., tank pressures). They note that these methods will be useful to oil and gas operators for near-term regulatory compliance as measurement-based methods for estimating emission durations evolve, such as more frequent aerial sampling^7,8^ or supplementing snapshot measurements with continuous monitoring systems (CMS).^9^

Here, we develop a method for estimating methane emission durations using point-in-space CMS. These sensor systems measure methane concentrations in near-real-time at several fixed sensor locations. In practice, 1 to 10 CMS sensors may be installed on a given site, with most production sites having around 4 sensors.

There are often times when wind blows emitted methane away from the CMS sensors, which we call *nondetect times*. During nondetect times, the sensors will not record elevated methane concentrations, making it incorrectly appear as if no emissions were occurring. In a simulated one-source scenario, Chen et al.^10^ find that nondetect times make up 78% of total time when using one CMS sensor and 45% of total time when using four CMS sensors. Nondetect times can cause a delay between emission onset and detection, ranging from 12 h on average using one sensor to 4.3 h on average using four sensors on a typical tank battery.^11^

In this work, we propose the Probabilistic Duration Model (PDM), a method for directly estimating methane emission durations using CMS that accounts for nondetect times. We evaluate the PDM using blinded controlled release data from the Methane Emissions Technology Evaluation Center (METEC). We then apply the PDM to CMS data collected on an oil and gas production site in the Appalachian basin as part of the Appalachian Methane Initiative (AMI) and use it to bound the duration of aerial measurements.

## Methods and Materials

First, we introduce a naive method for estimating emission durations that does not account for CMS nondetect times. Second, we introduce the PDM, which updates duration estimates from the naive method by probabilistically accounting for nondetect times. Third, we describe the controlled release data used to evaluate the PDM.

### Naive Method for Estimating Emission Durations

We use the method from Daniels et al.^12^ to create naive duration estimates. This method is based on the idea that elevated methane concentrations above the background likely indicate the presence of an emission. First, we take the minute-by-minute maximum across the concentration data from all CMS sensors on the site. This collapses the signal from each sensor into one time series that contains elevated concentrations at a given time if any of the sensors observed elevated concentrations at that time. Next, we apply the spike detection algorithm from Daniels et al.^12^ to this maximum value time series, which uses a gradient-based method to identify sharply elevated concentration values, or spikes. The spikes detected by this algorithm are clustered into groups, where spikes separated by less than 30 min are combined, and any resulting cluster of spikes less than 15 min long is discarded. The clusters of spikes are taken as the naive emission events in this study, which we refer to as *naive events*. *Naive durations* are simply the lengths of the naive events.

### The Probabilistic Duration Model (PDM)

The PDM is designed to improve the naive duration estimates described in the previous section by probabilistically accounting for CMS nondetect times. It does this by extending the duration of naive events and combining neighboring naive events within a Monte Carlo framework. The PDM is separated into four steps, which are described in the following subsections. A visual summary of the model is shown in Figure S1 in the Supporting Information (SI) file.

#### Characterize the Naive Events

We estimate an emission source and rate for each naive event using the method from Daniels et al.^12^ This allows us to more accurately quantify the CMS nondetect times in the following step. We estimate the emission source by comparing CMS concentration observations to forward simulated concentrations from each possible source. For each naive event, the estimated emission source is taken as the source whose simulated concentrations most closely match the actual concentration observations. We estimate an emission rate for each naive event by minimizing the mean squared error between simulated concentrations and CMS concentration observations over a range of possible emission rates. We use the Gaussian puff atmospheric dispersion model to forward simulate, which is described in detail in Section S2 of the Supporting Information and in Jia et al.^13^ This step imposes the assumption that each naive emission event has a single source.

#### Create Information Mask

We next identify the periods where we expect the wind to blow emitted methane toward the sensors (the CMS detect times, or *periods of information*) and between the sensors (the CMS nondetect times, or *periods of no information*). We do so for each naive event by simulating methane concentrations at the sensor locations using the estimated source and rate from the previous step. We simulate using the Gaussian puff model and wind data collected on the site. We then take the minute-by-minute maximum of the simulated concentrations across all sensors on the site and apply the spike detection algorithm from Daniels et al.^12^ to this maximum value time series. Clusters of spikes in the simulated concentrations are defined as periods of information as these are the times where a simulated emission event causes elevated concentrations at the sensor locations. Section S3 in the Supporting Information contains details about this step.

#### Compute Probability of Combining Naive Events

Occasionally, two or more consecutive naive events with the same source estimate are separated by a period of no information. There are two possible emission scenarios that could cause this situation: 1) the emission persisted through the period of no information, and 2) the emission stopped and a new emission started during the period of no information. We assume that naive events separated in this manner are more likely to be from the same emission if their estimated rates are similar, regardless of the length of the no information period.

We define the probability, , of combining a given naive event, *E*_*i*_, with another event, *E*_*j*_, as
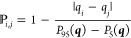
1where *q*_*i*_ and *q*_*j*_ are the estimated rates of events *E*_*i*_ and *E*_*j*_, and *P*_5_(***q***) and *P*_95_(***q***) are the 5th and 95th percentiles of all estimated rates. If *E*_*j*_ has a different source estimate than *E*_*i*_ or is separated by a period of information, then we set  Note that estimating emission frequency is relatively straightforward once  has been computed for each pair of naive events (see Section S4 in the Supporting Information for details).

#### Create Distribution of Possible Durations

We first identify the range of possible start and end times for each naive event without considering the probability of combining adjacent events. If a given naive event, *E*_*i*_, starts or ends during a period of information, then we assume there is only one possible start or end time for *E*_*i*_. However, if *E*_*i*_ starts at a transition from a period of no information to a period of information, then we assume all times back to the previous period of information are equally likely to be the start time of *E*_*i*_. Similarly, if *E*_*i*_ ends at a transition from a period of information to a period of no information, then we assume all times up to the next period of information are equally likely to be the end time of *E*_*i*_. See Figure S1 in the Supporting Information for a visual representation of this method.

We then use the following logic and Monte Carlo sampling to create a distribution of possible durations for naive event *E*_*i*_. We refer to *E*_*i*_ as the event that the PDM is “applied to”. If *E*_*i*_ has zero probability of being combined with either adjacent event, then we sample uniformly from the range of possible start and end times for *E*_*i*_. If *E*_*i*_ has nonzero probability of being combined with one adjacent event, *E*_*j*_, then we sample start times (if *E*_*j*_ comes before *E*_*i*_) or end times (if *E*_*j*_ comes after *E*_*i*_) with probability  from *E*_*j*_ and with probability 1 –  from *E*_*i*_. If *E*_*i*_ has nonzero probability of being combined with more than one adjacent event, then the procedure for sampling start and end times described above is applied recursively until an event, *E*_*k*_, with  is encountered (see Section S5 in the Supporting Information for details).

The differences between all combinations of sampled start and end times are taken as the distribution of possible durations for *E*_*i*_. A point estimate of the event duration can be produced by taking the mean or maximum (if an upper bound is desired) of this distribution. Note that the PDM can be used to bound the duration of a snapshot measurement by applying it to the naive event that coincides with the snapshot measurement.

### Controlled Release Evaluations

We use data from three controlled release experiments to evaluate the PDM: 1) the 2022 Advancing Development of Emissions Detection (ADED) campaign conducted at METEC,^14^ 2) the 2023 ADED campaign conducted at METEC,^15^ and 3) the 2022 Stanford high emission rate release campaign conducted in Arizona.^16^

For brevity, we show results from only the ADED 2023 campaign here, with results from the ADED 2022 and Stanford releases presented in the SI. The ADED 2023 campaign had 79 single-source controlled releases ranging from 0.01 to 7.1 kg/h in size and 0.02 to 9.0 h in duration. Methane concentration data for this evaluation came from 10 CMS sensors placed around the METEC facility. Our evaluation using the ADED 2023 data was conducted in a blinded manner. Sections S6–S8 in the Supporting Information contain a full description of the three controlled release campaigns and our procedure for blinding the data.

## Results

### Controlled Release Evaluations

**Figure 1:**
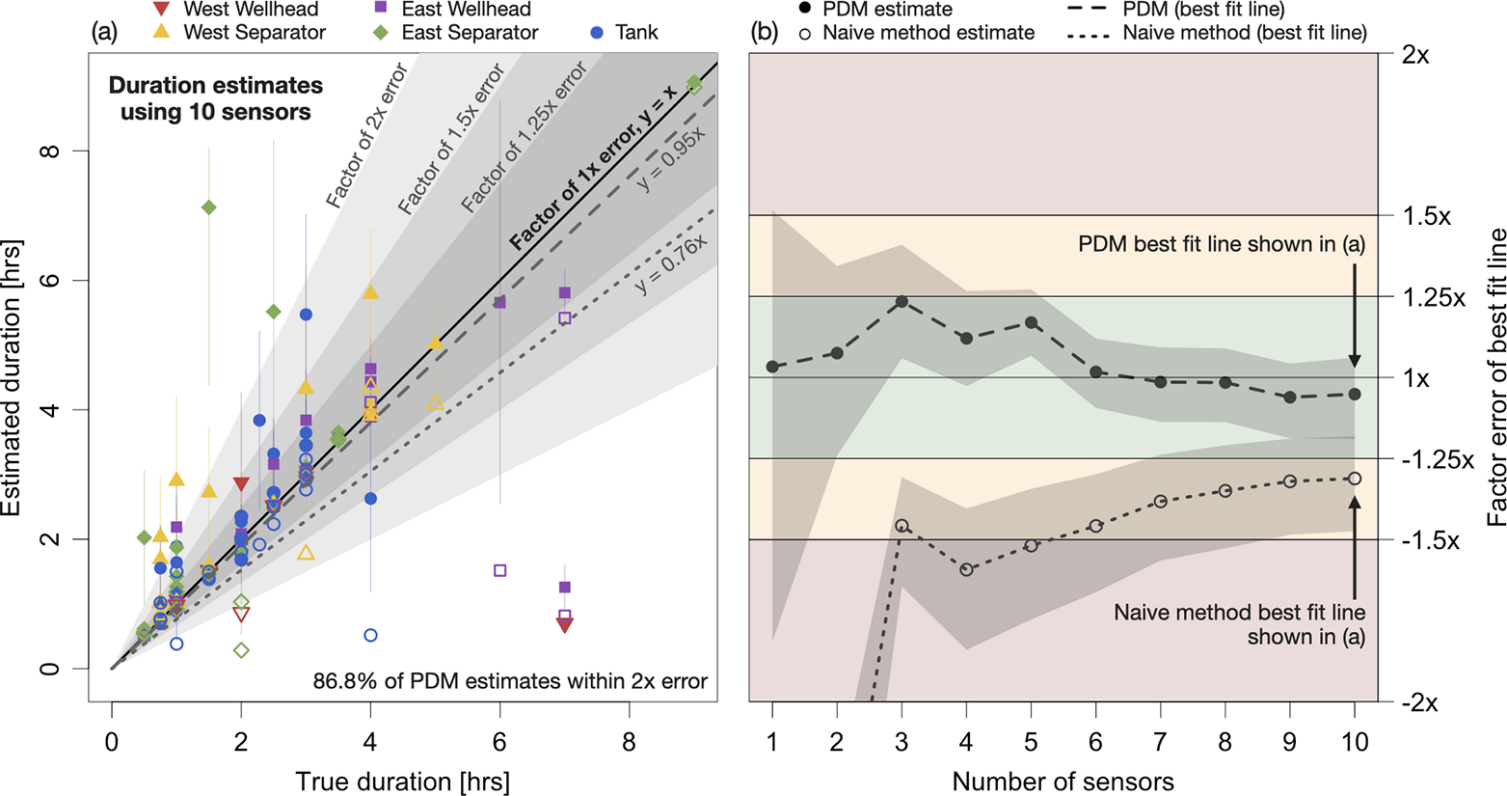
(a) Parity plot of estimated and true durations for the ADED 2023 controlled releases. Solid and empty points correspond to duration estimates from the PDM and naive methods, respectively, with vertical lines showing the 90% interval from the PDM and color showing the true emission source. Dashed and dotted lines show the best linear fit to the PDM and naive estimates, respectively. Gray shaded regions show three different error regimes. (b) Factor of over- or underestimation by the best linear fit to the PDM and naive estimates using different numbers of sensors. Gray shaded regions show the 95% confidence interval on the estimated slope. Negative factor differences indicate underestimation. Colored sections correspond to the three error regimes in (a). Note that the vertical scale is limited to [−2×, 2×] for visual clarity.

Figure 1 summarizes the performance of the naive method and the PDM on the blinded ADED 2023 controlled releases. Figure 1(a) compares duration estimates from both methods to the true durations using data from all 10 CMS sensors. We show duration estimates for events that coincide with a controlled release but not for false positive events, as there is no truth to compare these estimates against. The PDM estimates are taken as the mean of the possible durations provided by the model.

The slope of the best fit line to the PDM estimates is 0.95 (R^2^ = 0.80), indicating a slight tendency to underestimate the true durations. The naive method has a larger tendency to underestimate (slope = 0.76, R^2^ = 0.81), which makes sense for two reasons. First, CMS nondetect times often result in naive events that start too late or end too early. The PDM probabilistically extends these naive events by sampling start and end times from periods of no information. Second, CMS nondetect times often separate periods of elevated methane concentrations into multiple short naive events that each underestimate the duration of the coinciding release. The PDM probabilistically recombines these short events, resulting in more accurate duration estimates in the presence of CMS nondetect times.

The PDM’s benefit is more apparent when fewer CMS sensors are used, which is common in practice. To demonstrate this behavior, we recompute duration estimates using subsets of the 10 CMS sensors installed on the METEC site. For the *n*-sensor subset, we only use data from the *n* sensors that maximize detections by the sensor network based on wind data from the site. Figure 1(b) shows the degree of over- or underestimation by the best fit line for the naive method and PDM under different sensor subsets. While the PDM best fit line stays relatively constant within a factor of 1.25× error, the naive method best fit line decreases steadily as fewer sensors are used. This behavior is expected, as there are more opportunities for wind to blow emitted methane between sensors that are spaced farther apart. A similar analysis using suboptimal *n*-sensor arrangements is provided in Section S10 in the Supporting Information. Results from the ADED 2022 and Stanford releases are shown in Sections S6 and S8 of the SI file.

### Real Site Case Study

**Figure 2:**
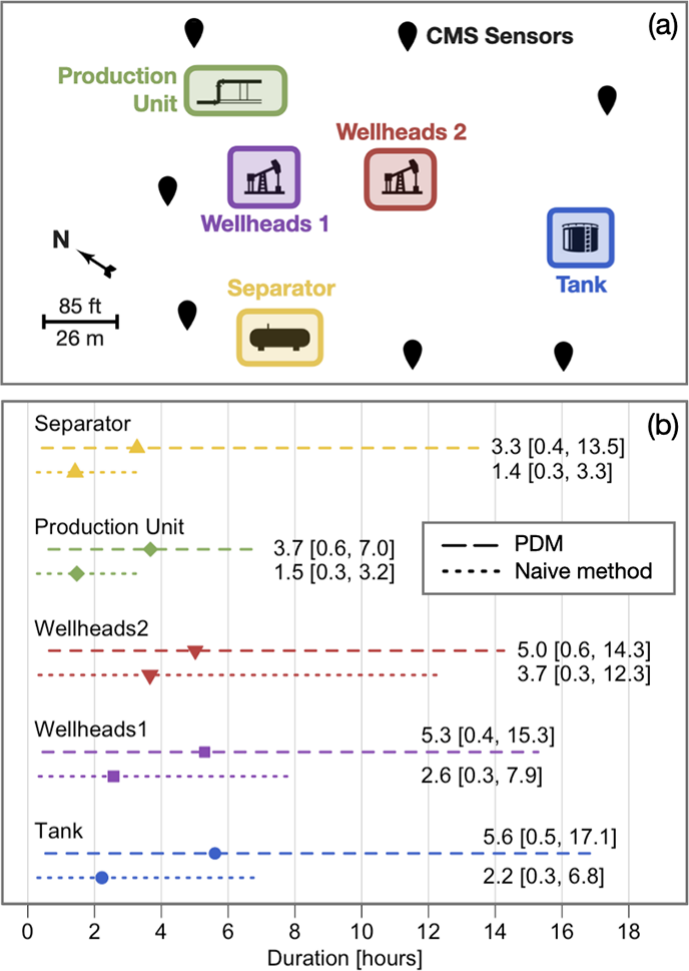
(a) Schematic of the oil and gas production site used as a case study in this article. (b) Summary of the duration estimates on this site. The left- and right-most points of the horizontal lines show the 5th and 95th percentiles of the duration estimates across all emission events. The marker symbols show the mean duration estimate. These summary statistics are printed on the figure in the following format: mean [5th percentile, 95th percentile].

We apply the PDM to CMS data collected from August 21 to October 31, 2023 on an oil and gas production site in the Appalachian basin. This site was selected for a case study because it has the simplest configuration among Appalachian Methane Initiative (AMI) sites instrumented with CMS and was, therefore, most likely to satisfy the single-source assumption of the PDM. Figure 2(a) shows a site schematic, and Figure 2(b) shows the range of naive duration estimates and PDM estimates across all identified emission events on the site. PDM estimates are taken as the mean of the possible durations provided by the model. Section S4 in the Supporting Information lists the emission frequency estimates for this site, and Sections S11–S13 provide additional details about the case study.

**Figure 3:**
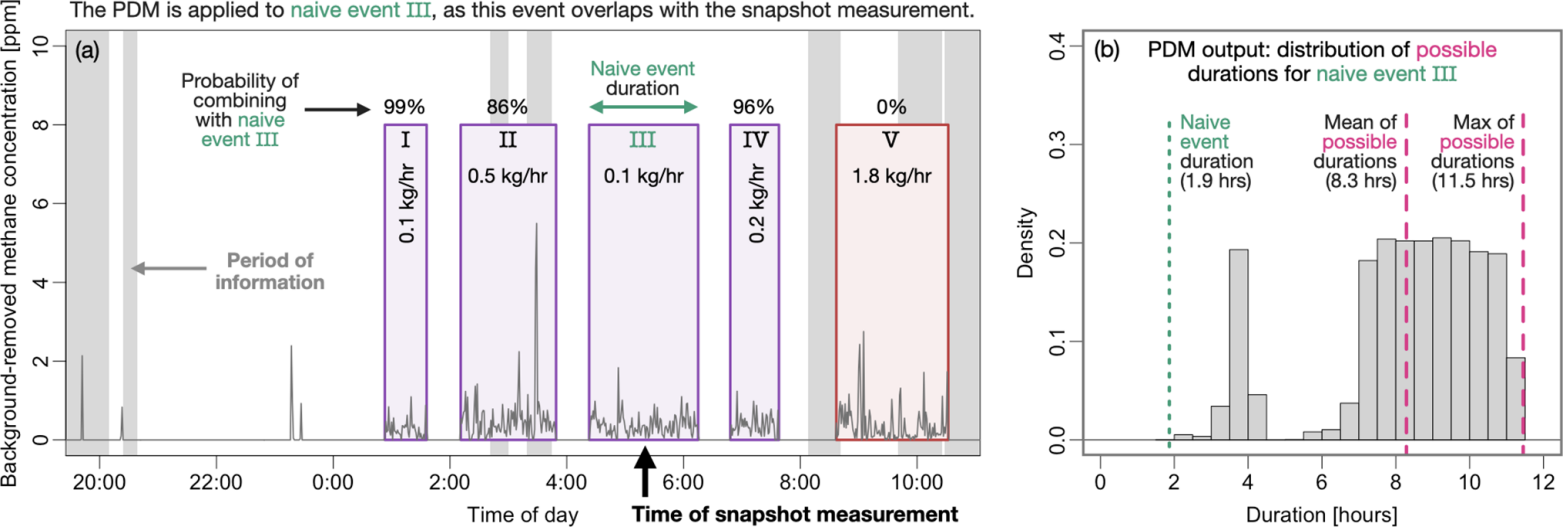
(a) Example snapshot measurement (time indicated by black arrow) and the overlapping CMS concentration data (spanning September 27, 2023 at 8:00 pm to September 28, 2023 at 10:30am). Enumerated boxes show the naive events, with the color indicating the source estimate (color corresponds to the schematic in Figure 2(a)). Gray shaded regions mark periods of information. Percents indicate the probability of combining each event with the event that overlaps the snapshot measurement. (b) Distribution of possible durations from the PDM for naive event III and correspondingly for the overlapping snapshot measurement.

We use the PDM to bound the duration of a hypothetical snapshot measurement on this site, as no actual snapshot measurements were taken while the CMS were deployed. Figure 3(a) shows the time of this hypothetical measurement and the overlapping CMS data. Without accounting for nondetect times, the duration of naive event III could be taken as the duration estimate for the coinciding snapshot measurement. However, there are multiple naive events also localized to Wellheads 1 surrounding event III, many of which are separated by periods of no information. Taking this into account via the PDM results in a distribution of possible emission durations for event III, shown in Figure 3(b), and hence a distribution of possible durations for the coinciding snapshot measurement. The naive duration estimate (1.9 h) is shorter than the mean (8.3 h) and maximum (11.5 h) estimates from the PDM by a factor of 4.4× and 6.1×, respectively. This underestimation would impact the estimate of the total emitted methane to the same degree.

To probe the extent of possible underestimation by the naive method, we repeat our analysis on this site for all possible snapshot measurement times. The largest instance of underestimation was by a factor of 36.4× and 64.8× compared to the mean and maximum estimates from the PDM, respectively.

Finally, we compare the PDM estimate to other methods for estimating emission duration. The previous Bridger overflight on this site occurred on May 17, 2023, and did not detect emissions from the Wellheads 1 equipment group, resulting in a survey-based duration estimate of 134 days. The emission event in Figure 3 is too small to be detected by satellites, so this measurement technology would be unable to estimate the duration of this event. If no measurements were conducted, the default duration for reporting to the EPA is 91 days.^1^

## Discussion

This study has revealed a number of important considerations for aerial measurement campaigns and the finalized EPA rule coming into effect in January 2025:

1. CMS can complement snapshot measurement technologies by bounding the duration of detected emissions. Survey-based aerial measurement campaigns are often performed only quarterly or yearly and hence have limited ability on their own to bound the duration of intermittent emission events.
2. If ignored, CMS nondetect times can result in significant underestimation of emission duration, to the point where the naive use of CMS for duration estimates could unintentionally circumvent a majority of the methane fees associated with large emissions. As such, addressing CMS nondetect times is critical for accurate duration estimates.
3. We propose a method for estimating emission durations using CMS that probabilistically accounts for nondetect times. The benefit of this method is especially apparent when only a small number of sensors are installed on a given site, which results in limited coverage of the site and is common in practice.

Current commercially available CMS solutions have large quantification errors on controlled releases,^14−16^ but their detection capabilities show promise, especially for larger emissions.^16^ Therefore, while their quantification capabilities evolve, CMS can complement snapshot measurement technologies by bounding the duration of detected emissions. Finally, we reiterate that the PDM assumes a single emission source for all detected emission events. This limits the accuracy of the PDM on complex sites where the single source assumption breaks down, as errors in the source location estimates will impact the accuracy of the information mask. Additional limitations of the PDM as currently implemented are discussed in Section S15 in the Supporting Information.

## Data Availability

Code and data are available at https://github.com/wsdaniels/CMS-durations.

## Supporting Information Available

Additional details about the controlled release experiment and the case study (PDF)
