# Supplementary material for: Estimating Methane Emission Durations Using Continuous Monitoring Systems
Source: Environ Sci Technol Lett. 2024 Oct 28;11(11):1187–92. doi: 10.1021/acs.estlett.4c00687 (PMC11562728; doi:10.1021/acs.estlett.4c00687)
Supplement: Supplementary file 1 — ez4c00687_si_001.pdf [file ez4c00687_si_001.pdf]

Supporting Information for:

Estimating methane emission durations using continuous monitoring systems

William S. Daniels[1], Meng Jia[1], and Dorit M. Hammerling[1,2]

[1]Department of Applied Mathematics and Statistics, Colorado School of Mines, Golden, Colorado 80401, United States

[2]Energy Emissions Modeling and Data Lab, The University of Texas at Austin, Austin, Texas 78712, United States

Email: wdaniels@mines.edu

Contents

S1 Visual summary of the Probabilistic Duration Model (PDM) 2

S2 The Gaussian puff atmospheric dispersion model 2

S3 Discussion and sensitivity analysis of PDM parameters 4

S4 Method for estimating emission frequency 5

S5 Additional details about computing the probability of combining naive events 6

S6 ADED 2022 controlled release experiment 7

S7 ADED 2023 controlled release experiment 8

S8 Stanford high emission rate controlled release experiment 9

S9 Comparison of blinded and non-blinded PDM performance 13

S10Effect of sensor placement optimization on duration estimates 13

S11Full detection, localization, and quantification results for AMI case study 17

S12Additional examples of bounding emission duration 19

S13Exhaustive search through all possible snapshot measurement times 20

S14Comparison of methods for estimating emission durations 21

S15PDM limitations and next steps 21

S1 Visual summary of the Probabilistic Duration Model (PDM)

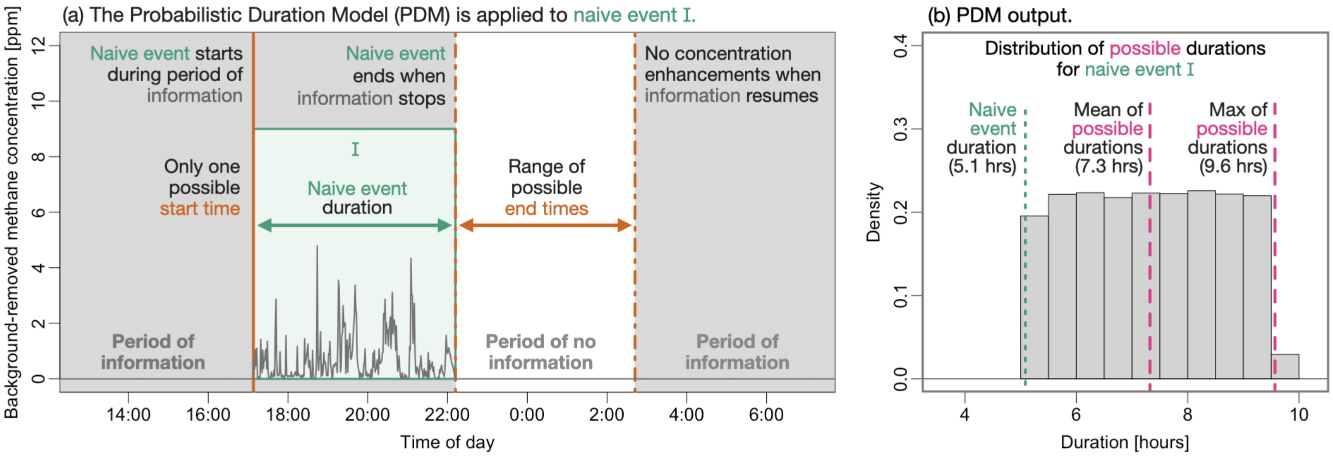

Figure S1: Visual summary of the Probabilistic Duration Model (PDM). (a) Concentration data from an example emission event. The identified naive event is highlighted by a green box, with the naive duration shown as an arrow. Periods of information are shaded in gray, while periods of no information have a white background. Possible start and end times based on the periods of information are marked in orange. (b) Result of applying the PDM to the naive event in (a).

S2 The Gaussian puff atmospheric dispersion model

This study uses the Gaussian puff atmospheric dispersion model to simulate the transport of methane through air. At a high level, the Gaussian puff model approximates a continuous release of some pollutant (in our case methane) by simulating the movement of many small "puffs" of methane that are modeled as 3-dimensional Gaussian-like distributions. The Gaussian puff model is used in the following steps of the Probabilistic Duration Model (PDM) described in the main text: 1) estimate naive event source location, 2) estimate naive event emission rate, 3) estimate periods of information.

Here we describe the Gaussian puff model in detail, its input parameters, and how we can tell when it is poorly approximating actual transport. Note that this description of the Gaussian puff model is a concise summary of the description provided in [1] and [2].

The first step of running the Gaussian puff model is to set the coordinate system so that the source is located at $(0, 0, H)$, where H is the height of the source. The "puffs" of methane simulated by the model will all originate at this point. New puffs are created at a given frequency, dt, by default once per second, and the coordinates are rotated for each puff so that the positive x-direction is pointed in the downwind direction at the time of puff creation. In this rotated coordinate system, the methane concentrations produced by a single puff, p, at sensor location (x, y, z) and time from creation t is modeled as

$$c_p(x, y, z, t) = \frac{Q}{(2\pi)^{3/2}\sigma_y^2\sigma_z} \exp\left(-\frac{(x-ut)^2+y^2}{2\sigma_y^2}\right)\left[\exp\left(-\frac{(z-H)^2}{2\sigma_z^2}\right)+\exp\left(-\frac{(z+H)^2}{2\sigma_z^2}\right)\right],$$

where Q is the mass of methane contained in puff p, σ_y and σ_z are the dispersion parameters in the y and z directions, respectively, and u is the wind speed at the time of puff creation. The value of Q is determined by the user specified emission rate, q, and the frequency of puff creation, dt.

At each subsequent time step, a new puff is created at the release point and puffs already in existence are advected based on the wind speed and direction at their respective creation times. This version of the Gaussian puff model assumes zero advection in the vertical direction, as wind data in this dimension are hard to obtain in practice. The total concentration at sensor location (x, y, z) and time t is then simply

$$c(x, y, z, t) = \sum_{p=1}^{P} c_p(x, y, z, t),$$

where P is the number of puffs in existence at time t.

The dispersion parameters (σ_y and σ_z) control the width of each puff. We obtain σ_y and σ_z values using the EPA parameterization of the Pasquill-Gifford-Turner dispersion scheme [3–5]. Under this parameterization, both σ_y and σ_z are functions of stability class and monotonically increase with distance traveled. This means that the puffs grow larger (i.e., more diffuse) over time as they move further away from the source.

In summary, the input parameters required to run this version of the Gaussian puff dispersion model are as follows:

- **Source locations.** Methane emission sources on oil and gas production sites are often well known and can be identified via satellite imagery or through discussion with the oil and gas operator.

- **Sensor locations.** CMS technology vendors always keep a log of where their sensors are located.

- **Wind speed and direction.** These data are almost always obtained by anemometers attached to the CMS sensors. High frequency wind data leads to better simulation fidelity. Ideally, the anemometers would obtain one observation of wind speed and direction per second.

- **Emission rate, q.** The value of this parameter depends on the use-case of the Gaussian puff model. When estimating the emission source and rate of each naive event, the emission rate provided to the Gaussian puff model does not matter. This is because the output of the Gaussian puff model is a linear function of emission rate, and the method for estimating emission source and rate (described in detail in [1]) does not depend on the amplitude of the simulated concentrations (only their temporal pattern). Therefore, an arbitrary value of 1 g/s is used when estimating the emission source and rate for each naive event. When using the Gaussian puff model to identify the periods of information, however, the overall amplitude of the simulated concentrations does matter. This is because large enhancements in the simulated concentration time series (defined as a background-correct amplitude of 0.75 ppm or larger) are taken as periods of information. Therefore, when using the Gaussian puff model to identify periods of information for a given naive event, we run the simulation using the estimated emission rate for that naive event. This scales the simulated concentrations to our best estimate of the true emission rate during the naive event.

- **Puff creation frequency, dt.** Higher simulation frequencies result in more accurate output, as there is more overlap between the puffs and hence a better approximation of a continuous release. However, simulating more puffs per second increases the computational cost of the dispersion model. This parameter is set to 1 puff per second by default, which has been found to be more than adequate fidelity for this application [2].

- **Dispersion parameters, σ_y and σ_z.** These values are obtained from a look-up table contained in [5].

Finally, we do not produce an emission rate estimate for a given naive event if we believe that the Gaussian puff atmospheric dispersion model is not accurately representing actual transport during that naive event. The Gaussian puff model may poorly approximate actual transport if there is a high degree of turbulence, which is not included in the Gaussian puff model, or if the real methane plume is obstructed by a building or large piece of equipment, as these types of blockage are also not included in the Gaussian puff model. For each naive event, we test if the Gaussian puff model is decently approximating actual transport by counting the number of enhancements in the simulated concentration time series that temporally align with enhancements in the CMS concentration observations. If there are more than 4 instances of alignment, we say that the Gaussian puff model is providing a good enough approximation of reality to estimate an emission rate. See [1] for a deeper discussion of this alignment threshold.

S3 Discussion and sensitivity analysis of PDM parameters

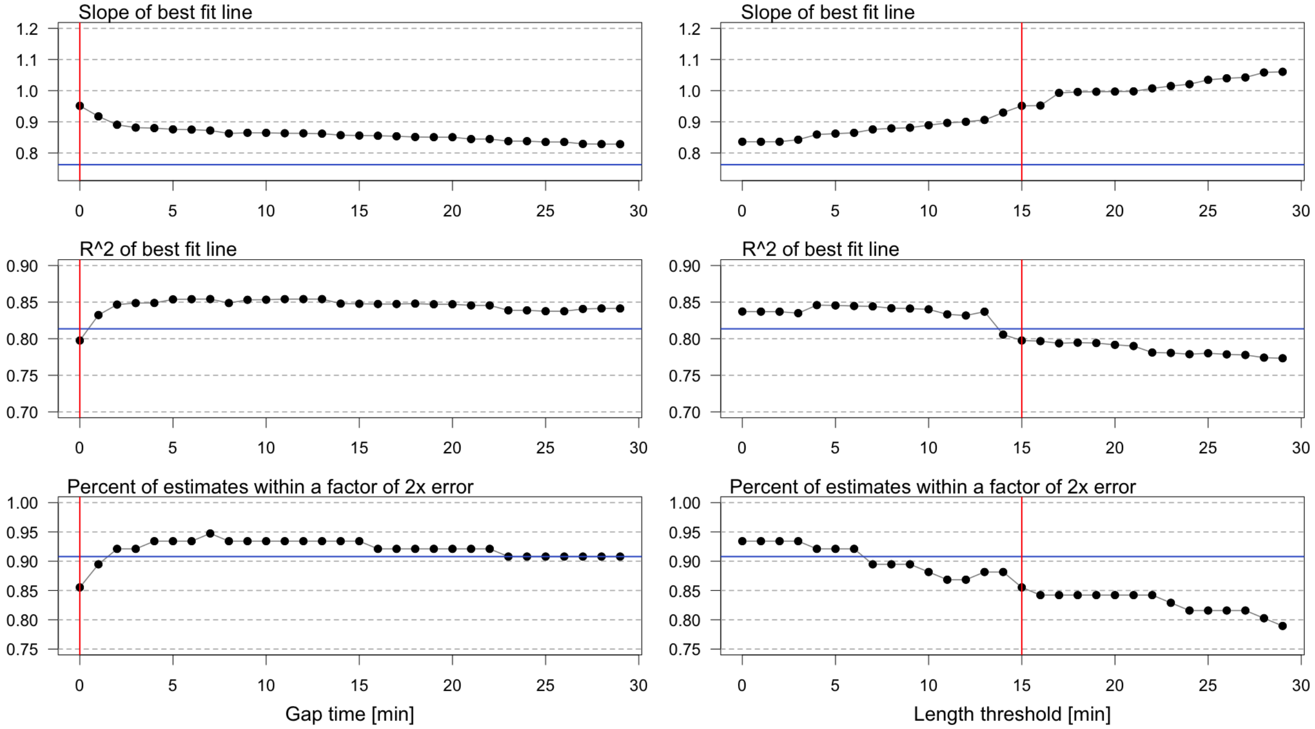

Figure S2: Sensitivity of the PDM output to the fixed parameters that control the degree of smoothing of the information mask. This sensitivity study was performed on the ADED 2023 controlled release data and only analyzes PDM output using all 10 CMS sensors. Vertical red lines show the default values of the two parameters. When one parameter is varied, the other is left at its default value. Horizontal blue lines show the value obtained using the naive method. “Slope of best fit line” is the slope of the line fit to the duration estimates and the true durations, with values less than 1 indicating underestimation. “R^2 of best fit line” is the R^2 value of the same best fit line. “Percent of estimates within a factor of 2x error” is the percent of duration estimates that are within a factor of 2x error from the true duration.

The PDM has two fixed parameters that control the amount of smoothing applied to the information mask: the gap time and the length threshold. Both are parameters of the spike detection algorithm from [1] that is used to identify and cluster spikes in the simulated concentration time series. These clusters of spikes are what determine the periods of information or no information.

Spikes in the simulated concentration time series are combined if they are separated by “gap time” minutes or less. This parameter can be used to smooth out short periods of time where the wind is blowing away from the sensors, but then returns quickly. The default value for this parameter is 0 minutes, as we have found that even extremely short periods of time when wind is blowing away from the sensors results in CMS non-detect times.

Clusters of spikes in the simulated concentration time series are discarded if their length is less than “length threshold” minutes. This effectively removes short periods of enhancements that may be due to noisy data or errors in the forward dispersion model. Occasionally, there will be large jumps in the wind data that result in a brief spike in the simulated concentrations that is not seen in the actual concentration observations, and hence these short-lived spikes are removed. This can again be thought of as smoothing out the information mask, as it removes short bursts of information that are likely not realistic.

A sensitivity study of these two parameters is provided in Figure S2 using data from the ADED 2023 evaluation. Note that the ADED 2022 evaluation, not the ADED 2023 evaluation, was used to set the default values for these parameters. When the gap time is increased from its default value of zero minutes, all of the metrics trend toward

their value when using the naive method. This makes sense, as increasing the gap time effectively eliminates short periods of no information. In the limit when all of these periods are eliminated, the PDM returns the same duration estimates as the naive method. When the length threshold is decreased from its default value of 15 minutes, the slope of the best fit line decreases (i.e., the PDM duration estimates are shorter), and vice versa. This again makes sense, as a shorter length threshold means that fewer clusters of spikes in the simulated concentration data are discarded, and as a result, there are fewer periods of no information. When there are fewer periods of no information, the event start and end times that are sampled by the PDM are closer to the start and end times of the naive events, and hence the durations from the PDM are shorter. The opposite is true as well. When the length threshold is increased, more clusters of spikes in the simulated concentration data are discarded, and as a result, there are more periods of no information. This means that the start and end times sampled by the PDM are further from the start and end times of the naive events, and hence the durations from the PDM are longer.

These results are promising, as we set the default parameters using completely different data (i.e., the data from the 2022 ADED evaluation). Despite this, when evaluated on the new ADED 2023 data, the default values still result in close to optimal model performance. That is, the default values result in best fit slopes close to parity.

Note that we do not include the precision of the methane sensors as a fixed parameter of the PDM, as current commercially available CMS solutions with quantification capabilities have very precise instrumentation (on the order of 1 ppm or less). Therefore, it is very unlikely that measurement error from the methane sensors will impact the PDM results.

S4 Method for estimating emission frequency

Recall that we define the probability of combining a given naive event, E_i, with a neighboring event, E_j, as

$$\mathbb{P}_{i,j} = 1 - \frac{|q_i - q_j|}{P_{95}(\boldsymbol{q}) - P_5(\boldsymbol{q})}, \tag{1}$$

where q_i and q_j are the estimated emission rates of naive events E_i and E_j, $\boldsymbol{q}$ is a vector of estimated emission rates for all naive events, and $P_5()$ and $P_{95}()$ are functions returning the 5th and 95th percentiles.

Once $\mathbb{P}_{i,j}$ has been computed for each pair of naive events, then estimating emission frequency within a given time interval is straightforward. To do so, we simply count the number of naive events per emission source within the given time interval, combining them with their neighbors with probability $\mathbb{P}_{i,j}$. If two naive events are combined, they are only counted as one event in the emission frequency calculation. This process is repeated many times within a Monte Carlo framework to provide many realizations of the possible event combination options, resulting in a distribution of possible counts for each emission source. These distributions are then annualized to provide source-level frequency estimates.

We now provide frequency estimates for the site shown in Figure 2(a) of the main text using the method described above. The average number of emissions per year per source and 90% intervals are as follows: 235 [231, 241] production unit emissions per year, 423 [416, 431] wellheads1 emissions per year, 87 [87, 87] wellheads2 emissions per year, 256 [246, 262] separator emissions per year, and 390 [374, 405] tank emissions per year.

S5 Additional details about computing the probability of combining naive events

Recall that we define the probability, $\mathbb{P}_{i,j}$, of combining a given naive event, E_i, with another event, E_j, as

$$\mathbb{P}_{i,j} = 1 - \frac{|q_i - q_j|}{P_{95}(\boldsymbol{q}) - P_5(\boldsymbol{q})}, \tag{2}$$

where q_i and q_j are the estimated emission rates of naive events E_i and E_j, $\boldsymbol{q}$ is a vector of estimated emission rates for all naive events, and $P_5()$ and $P_{95}()$ are functions returning the 5th and 95th percentiles.

If it is not possible to produce a rate estimate for E_i or E_j, then we set $\mathbb{P}_{i,j} = 0.5$, which assigns equal probability of combining and not combining the two events. A rate estimate is not produced when we determine that the Gaussian puff atmospheric dispersion model is poorly representing actual atmospheric transport during a given event. This determination is made by thresholding the degree of alignment between the simulated concentrations and the CMS concentration observations (see Section S2 of the SI for details on the Gaussian puff atmospheric dispersion model).

We now describe the recursive sampling strategy used when more than two adjacent naive events have non-zero probability of being combined. To describe this algorithm, assume that we apply the PDM to naive event E_1, which has non-zero probability of being combined with the two subsequent naive events, E_2 and E_3. While this example only contains three events, the same logic can be applied to a situation with n many events without loss of generality.

First, we compute $\mathbb{P}_{1,2}$ and $\mathbb{P}_{1,3}$ using Equation 2, which is reproduced from the main text. Using this equation, $\mathbb{P}_{1,2}$ and $\mathbb{P}_{1,3}$ can be thought of as the probability of directly combining E_1 with either E_2 or E_3 without considering the fact that E_2 comes between E_1 and E_3. However, we impose the assumption that E_1 and E_3 can only be combined in a given iteration of the Monte Carlo framework if E_1 is also combined with E_2 during that same iteration. Therefore, we must compute an overall probability of combining E_1 with E_3 that takes into account the probability of combining E_1 with the intermediate event E_2. As a first step in doing so, we compute the cumulative product of the probabilities of combining E_1 with the adjacent events. This results in the following vector:

$$\{C\mathbb{P}_1,\ C\mathbb{P}_2,\ C\mathbb{P}_3\} = \{1,\ \mathbb{P}_{1,2},\ \mathbb{P}_{1,2}\mathbb{P}_{1,3}\}.$$

These values can be thought of as the probabilities of combining E_1 with either itself, E_2, or E_3, accounting for the fact that E_1 can only be combined with E_3 in a given sample if it is also combined with E_2. The final step of this algorithm incorporates the fact that if there is non-zero probability of combining E_1 with E_2 and E_3, then there is no longer 100% probability of drawing an end time sample for E_1 from only the range of possible end times for E_1. We do this by recursively subtracting the cumulative probabilities from one another, starting with the last possible event, E_3, and working back towards E_1. This results in the normalized vector of probabilities

$$\{C\mathbb{P}_1 - (C\mathbb{P}_2 - C\mathbb{P}_3) - C\mathbb{P}_3,\ C\mathbb{P}_2 - C\mathbb{P}_3,\ C\mathbb{P}_3\},$$

which sum to 1 and are the final probability values used to sample end times for E_1. In other words, when sampling end times for E_1 in this example, there is a $C\mathbb{P}_1 - (C\mathbb{P}_2 - C\mathbb{P}_3) - C\mathbb{P}_3$ percent chance of drawing the sample from the possible end times of E_1, a $C\mathbb{P}_2 - C\mathbb{P}_3$ percent chance of drawing the sample from the possible end times of E_2, and a $C\mathbb{P}_3$ percent chance of drawing the sample from the possible end times of E_3.

To make this example more concrete, let us now use hypothetical numbers. Let $\mathbb{P}_{1,2} = 0.8$ and $\mathbb{P}_{1,3} = 0.6$. This gives us the cumulative probabilities

$$\{C\mathbb{P}_1,\ C\mathbb{P}_2,\ C\mathbb{P}_3\} = \{1,\ 0.8,\ 0.48\},$$

and ultimately a probability of sampling end times for E_1 from naive events E_1, E_2, and E_3 of

$$\{0.2,\ 0.32,\ 0.48\},$$

respectively. Note that this example involved combining a naive event with multiple adjacent events that come later in time. The same logic is applied to situations where the adjacent events come earlier in time and the situations where a naive event is combined with multiple events both preceding and following it.

S6 ADED 2022 controlled release experiment

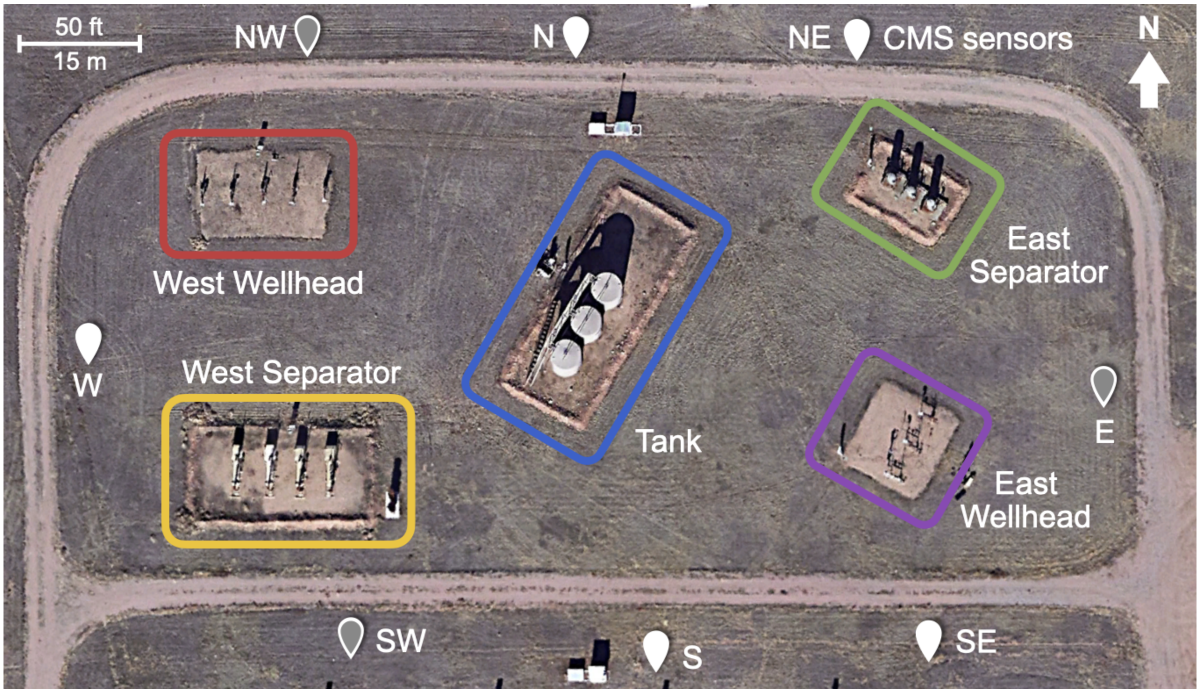

Figure S3: Satellite imagery of the Methane Emissions Technology Evaluation Center (METEC) during the ADED 2022 experiment. Potential emission sources are marked with colored boxes. CMS sensor locations are marked with white pins and are denoted by their cardinal direction relative to the site. Pins with a gray interior indicate sensors that measure wind speed and direction in addition to methane concentrations.

We performed a preliminary evaluation of the PDM on non-blinded controlled releases conducted as a part of the 2022 Advancing Development of Emissions Detection (ADED) research program at the Methane Emissions Technology Evaluation Center (METEC) in Fort Collins, Colorado.

We filtered the controlled releases to single-source releases only, as the PDM assumes a single emission source. In doing so, we focused on roughly one month of the experiment, from April 17, 2022 to May 15, 2022, which contained primarily single source releases. Any multi-source releases in this time frame were discarded, along with any naive emission events that overlapped with the multi-source releases. This filtering included naive events that were probabilistically recombined with neighboring naive events that overlapped with a multi-source release, as including these events would artificially inflate the duration estimates from the PDM.

This filtering resulted in 85 single-source emissions for the 8-sensor case. Releases ranged from 0.50 to 8.25 hrs in duration and 0.18 to 6.39 kg/hr in size. Emissions could come from one of five potential emission sources on the METEC site, marked with colored boxes in Figure S3.

The continuous monitoring system (CMS) sensors used in this evaluation are from Project Canary [6] and are shown with white pins in Figure S3. Pins with a gray interior indicate sensors that measure wind speed and direction in addition to methane concentrations. The CMS sensors were installed at a height of 2.4 m. Project Canary used Near-IR Tunable Diode Laser Absorption Spectroscopy (TDLAS) methane sensors during these releases. These sensors have an accuracy of ±2% and a precision of ≤ 0.125 ppm with 60 s averaging as reported by the manufacturer (R. Mistry, personal communication, January 17, 2024). The anemometers used by Project Canary have an accuracy of ±2% ±0.3 m/s for wind speed and ±2 degrees for wind direction and a resolution of 0.01 m/s for wind speed and 0.1 degrees for wind direction as reported by the manufacturer [7].

Results of the ADED 2022 experiment are shown in Figure S4 using the same parity plot structure as Figure 1 in the main text. We used the ADED 2022 evaluation to determine default values for the two fixed parameters

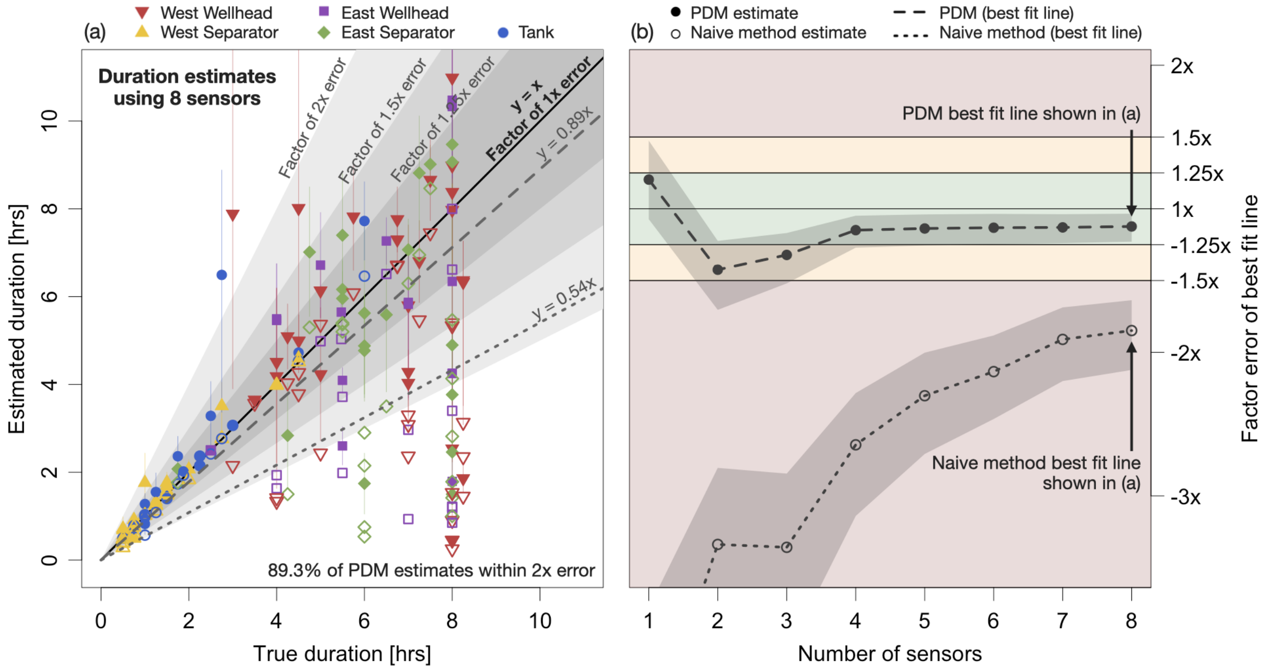

Figure S4: (a) Parity plot of estimated and true durations for the ADED 2022 controlled releases. Solid and empty points correspond to duration estimates from the PDM and naive methods, respectively, with vertical lines showing the 90% interval from the PDM and color showing the true emission source. Dashed and dotted lines show the best linear fit to the PDM and naive estimates, respectively. Gray shaded regions show three different error regimes. (b) Factor of over or underestimation by the best linear fit to the PDM and naive estimates using different numbers of sensors. Gray shaded regions show the 95% confidence interval on the estimated slope. Negative factor differences indicate underestimation. Colored sections correspond to the three error regimes in (a). Note that the vertical scale is limited to [-3.5x, 2x] for visual clarity.

of the PDM. These parameters control the degree of smoothing applied to the information mask and are discussed in detail in Section S3. To avoid overfitting these parameters, we did not perform an exhaustive search through their parameter space, but rather picked values that made intuitive sense and resulted in a visually well smoothed information mask.

S7 ADED 2023 controlled release experiment

We performed a more robust evaluation of the PDM on blinded controlled releases conducted as a part of the 2023 ADED research program at METEC in Fort Collins, Colorado. This experiment spanned from February 2, 2023 to April 28, 2023. The data were blinded using the following procedure. First, we split the author team into blinded and non-blinded groups. The non-blinded group acquired the true release information from METEC, while the blinded group acquired the raw concentration data from the CMS technology vendor. Next, without accessing the true release information, the blinded group ran the PDM on the raw CMS concentration data and finalized the results. After finalizing the PDM results, both the true release information and the PDM results were shared among the entire author team.

After finalizing the PDM results and sharing the ground truth data among the full author team, we then removed all multi-source releases from the truth data, along with any overlapping duration estimates in the finalized PDM results. This was done because the PDM assumes a single emission source at any point in time, so we only evaluate it on these scenarios. This filtering included naive events that were probabilistically recombined with

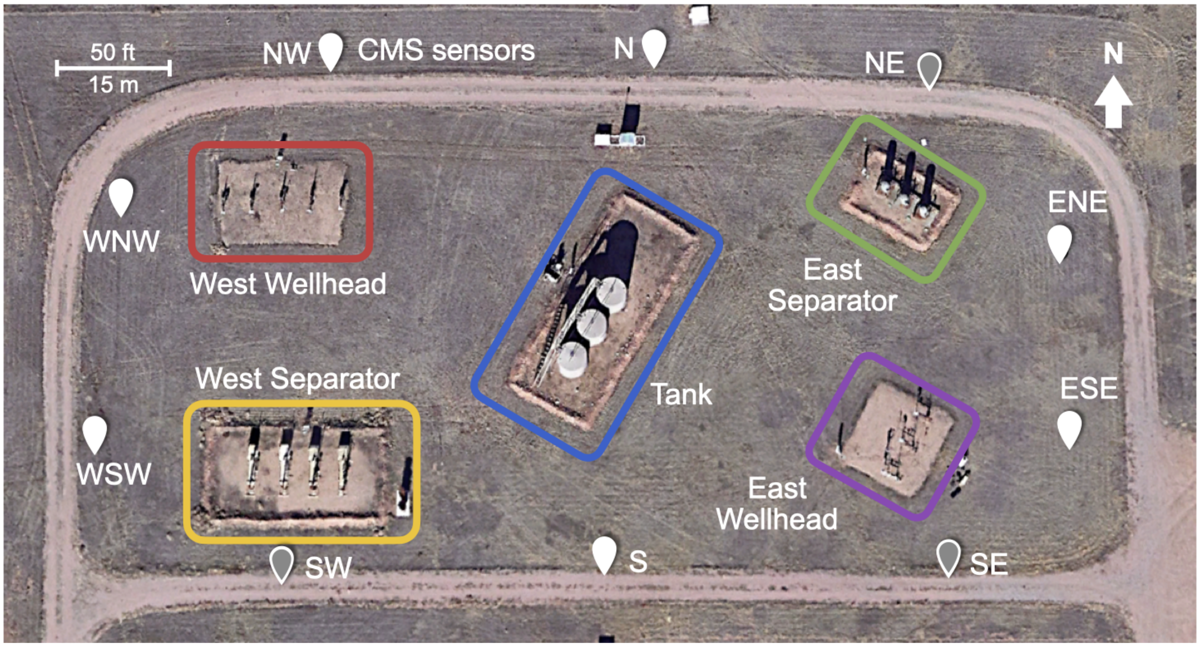

Figure S5: Satellite imagery of the Methane Emissions Technology Evaluation Center (METEC) during the ADED 2023 experiment. Potential emission sources are marked with colored boxes. CMS sensor locations are marked with white pins and are denoted by their cardinal direction relative to the site. Pins with a gray interior indicate sensors that measure wind speed and direction in addition to methane concentrations.

neighboring naive events that overlapped with a multi-source release. Including these events would artificially inflate the duration estimates from the PDM, as the events that overlapped with the multi-source releases would not have existed if the multi-source releases were not conducted. We also discarded one controlled release on March 6, 2023 and the corresponding duration estimate, as it was from a source not included in the study (i.e., not one of the five sources shown in Figure S5).

This filtering resulted in 79 single-source emissions for the 10 sensor case. Releases ranged from 0.02 to 9.0 hrs in duration and 0.01 to 7.1 kg/hr in size. Emissions could come from one of five potential emission sources on the METEC site, marked with colored boxes in Figure S5.

The continuous monitoring system (CMS) sensors used in this evaluation are from Project Canary [6] and are shown with white pins in Figure S5. Pins with a gray interior indicate sensors that measure wind speed and direction in addition to methane concentrations. The CMS sensors were installed at a height of 2 m. Project Canary used Near-IR Tunable Diode Laser Absorption Spectroscopy (TDLAS) methane sensors during these releases. These sensors have an accuracy of ±2% and a precision of ≤ 0.125 ppm with 60 s averaging as reported by the manufacturer (R. Mistry, personal communication, January 17, 2024). The anemometers used by Project Canary have an accuracy of ±2% ±0.3 m/s for wind speed and ±2 degrees for wind direction and a resolution of 0.01 m/s for wind speed and 0.1 degrees for wind direction as reported by the manufacturer [7].

Results of the ADED 2023 experiment are shown in Figure 1 in the main text.

S8 Stanford high emission rate controlled release experiment

We performed a third evaluation of the PDM on blinded controlled releases conducted in Arizona by Stanford University. This experiment spanned from October 10, 2022 to November 30, 2022. Our procedure for blinding these data is the same as the procedure used for the ADED 2023 evaluation. The Stanford controlled releases had only a single emission source, so no filtering was required to satisfy the single source assumption of the PDM.

This experiment had 107 single-source emissions. Before any data filtering, the releases ranged from 0.02 to

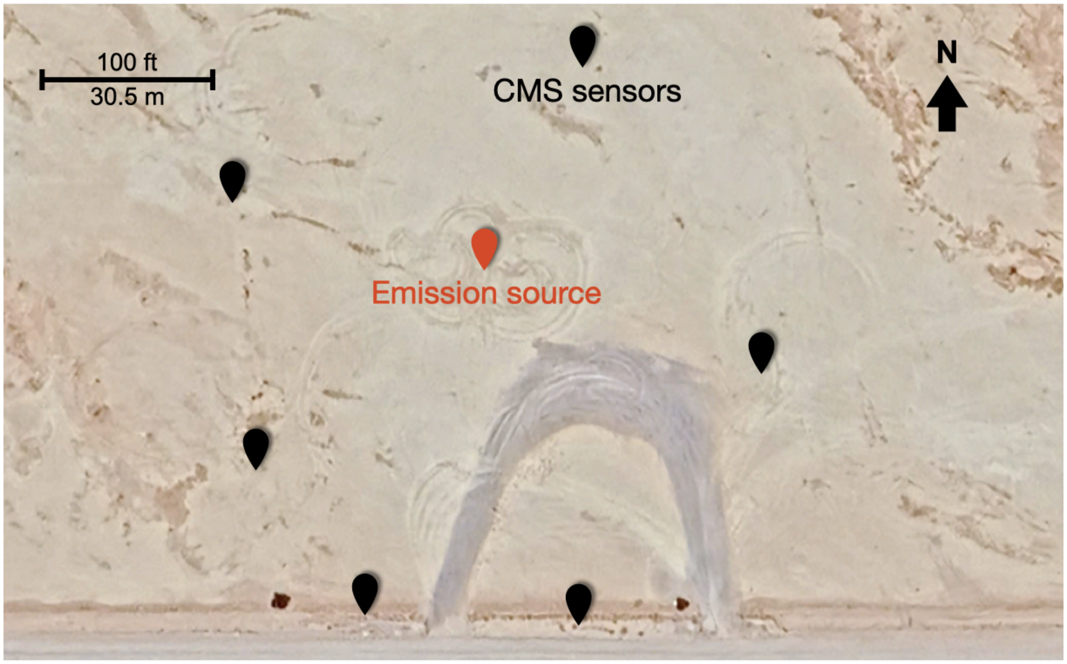

Figure S6: Satellite imagery of the location of the 2022 Stanford high emission rate controlled releases. The single emission source is marked with a red pin. CMS sensor locations are marked with black pins.

4.14 hrs in duration and 0.8 to 1363.6 kg/hr in size. Emissions could come from one central release point, marked with a red pin in Figure S6, at either a height of 3.0 m or 7.3 m. The Stanford releases were conducted such that there were often very short periods of no emissions between two releases at different emission rates. Specifically, there were 10 gaps less than 1 minute long, 44 gaps less than 5 minutes long, 56 gaps less than 10 minutes long, and 66 gaps less than 15 minutes long. Neither the PDM nor the naive method discussed in the main text were designed to identify periods of no emissions this short. In other words, two clusters of concentration enhancements separated by a gap of 15 minutes or less are assumed to be the same emission event by both the PDM and the naive method. Therefore, for the purpose of evaluating these methods, we combine the controlled releases that are separated by 15 minutes or less. When combining controlled releases, we average the emission rates across the releases that are being combined. Without taking this step, there would be 20 identified naive events that overlap with more than one controlled release (because the controlled releases are separated by only a few minutes). This case (i.e., one naive event overlapping with multiple controlled releases) is currently not plotted in the parity plot structure used throughout the article. There are no cases of this happening with the ADED 2022 and ADED 2023 data. Combining the controlled releases in this way results in 41 single-source controlled releases. The combined releases ranged from 0.2 to 6.8 hrs in duration and 9.0 to 1363.6 kg/hr in size. There were 25 releases with emission rate greater than the EPA's 100 kg/hr reporting threshold.

The CMS sensors used in this evaluation are from QUBE Technologies [8] and are shown with black pins in Figure S6. All of the 6 CMS sensors measured wind speed and direction in addition to methane concentrations. The CMS sensors were installed at a height of 2.2 m. QUBE used metal oxide methane sensors during these releases. These sensors have an accuracy of $\pm 1\%$ and a precision of 1 ppm as reported by the manufacturer (E. Wen, personal communication, September 27, 2024). The anemometers used by QUBE have a wind speed resolution of 1 km/hr and a wind direction resolution of 1 degree as reported by the manufacturer (E. Wen, personal communication, September 27, 2024), where resolution is defined as the smallest change in a quantity that can be detected by the sensor.

Results of the Stanford experiment are shown in Figure S7 using the same parity plot structure as Figure 1 in the main text. Both the PDM and the naive method exhibit a larger tendency to underestimate when evaluated on the 2022 Stanford high emission rate releases. When all 6 CMS sensors are used, the slope of the best fit line to

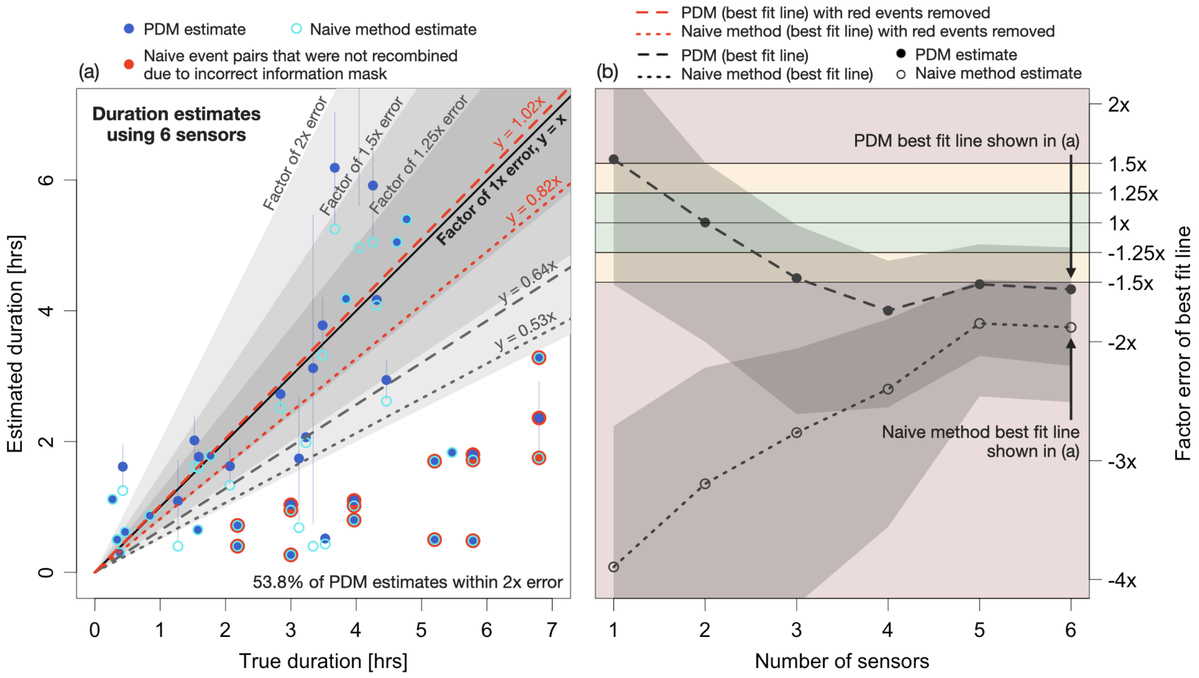

Figure S7: (a) Parity plot of estimated and true durations for the Stanford controlled releases. Solid and empty points correspond to duration estimates from the PDM and naive methods, respectively, with vertical lines showing the 90% interval from the PDM. Dashed and dotted lines show the best linear fit to the PDM and naive estimates, respectively. Points with red outlines are naive event pairs that occurred during one controlled release but were not recombined by the PDM. Gray shaded regions show three different error regimes. (b) Factor of over or underestimation by the best linear fit to the PDM and naive estimates using different numbers of sensors. Gray shaded regions show the 95% confidence interval on the estimated slope. Negative factor differences indicate underestimation. Colored sections correspond to the three error regimes in (a).

the PDM estimates is 0.64 (R^2 = 0.56, factor error = -1.56x) and the slope of the best fit line to the naive estimates is 0.53 (R^2 = 0.63, factor error = -1.88x). Almost all cases of extreme underestimation by the naive method (factor error less than -2x) were the result of one controlled release being separated into two short naive events due to a gap in the period of elevated methane concentrations. In these cases, we include the two short duration estimates in the parity plot and attribute both to the same true duration that is much longer. This noticeably decreases the slope of the best fit line. These events are outlined in red in Figure S7.

Unlike the ADED 2022 and 2023 experiments, the PDM did not recombine any of these naive event pairs, as almost all of them were erroneously separated by periods of information (resulting in zero probability of being combined). When these naive event pairs and the corresponding PDM estimates are removed, the slope of the best fit line to the PDM estimates increases to 1.02 (R^2 = 0.74, factor error = 1.02x) and the slope of the best fit line to the naive estimates increases to 0.82 (R^2 = 0.80, factor error = -1.22x). These best fit lines are shown in red in Figure S7.

There are two potential causes for the PDM failing to recombine the naive event pairs that overlap with one controlled release. First, the information mask was correct and there were no enhancements in the methane concentration measurements between the two naive events, despite wind blowing from the source to the sensors. Second, the information mask was incorrect, and the sensors measured no concentration enhancements because of CMS non-detect times (i.e., the wind was blowing the methane away from the sensors). The first of these two options is unlikely, as the concentration enhancements during the naive event pairs were often very large (between 20 and 60 ppm above background). Therefore, it is unlikely that the sensors failed to separate background concentrations from the methane plume during the middle of these controlled releases, as there was clear separation between these two signals during the time periods of the two naive events. Figure S8 shows an example of two naive events that

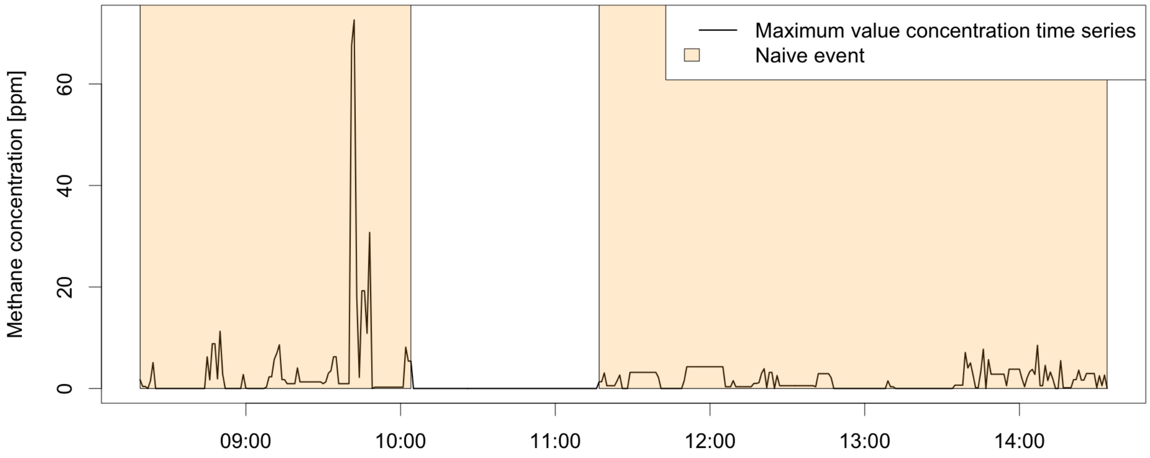

Figure S8: Example showing two naive events that overlap with one controlled release. Black line shows the minute-by-minute maximum taken across the concentration observations from all CMS sensors on the site. Orange boxes show the time periods of the naive events.

overlapped with one controlled release. Due to the size of the concentration enhancements during the naive event pairs, it is more likely that the information mask was incorrect and the naive event pairs were actually separated by CMS non-detect times.

A likely cause of the incorrect information mask is the presence of plume rise. We use the Gaussian puff atmospheric dispersion model to estimate the periods of information and no information, and this model does not account for plume rise. Therefore, if in reality the methane plume rose notably from the release point, there would be important differences in the concentration observations from the CMS sensors and the simulated concentrations from the Gaussian puff model.

The relative heights of the sources and sensors matter when considering the specific impact of plume rise. In this experiment, the release point was at a height of either 3.0 m or 7.3 m, while the sensors were all installed at a height of 2.2 m. This means that the release points were always higher than the CMS sensors. Therefore, if there was plume rise, the actual methane plume would have been farther away from (i.e., higher than) the CMS sensors than the simulated plume from the Gaussian puff model, which assumes zero plume rise. This in turn would have resulted in larger simulated concentrations at the sensor locations than if the Gaussian puff model had incorporated plume rise. Since the information mask is a function of the amplitude of the simulated concentrations at the sensor locations, this means that there could have been incorrect estimates of information due to the larger simulated concentrations. In other words, the Gaussian puff model may have missed the CMS non-detect times between these naive event pairs because it simulated the methane plume too low, closer to the CMS sensors than the actual methane plume. Note that this effect could also be contributing to the high degree of underestimation in the emission rate estimates from the technology vendors in Chen et al. [9].

There are two potential reasons why plume rise had a larger impact on the Stanford releases than the ADED 2022 and 2023 releases at METEC. First, the release point in the Stanford experiment was facing upwards, while there is potentially more variability in the direction of the release point at METEC. This would have introduced vertical velocity into the emitted methane during the Stanford releases. Second, the higher emission rate releases potentially further increased the vertical velocity of the methane at the release point. If this was the case, then it makes sense that this affected the Stanford releases more than the METEC releases, as the emission rates used in the Stanford releases were much larger than what is possible at METEC.

S9 Comparison of blinded and non-blinded PDM performance

Table 1 shows a general comparison of PDM performance on the two METEC experiments to highlight similarities and differences between the blinded and non-blinded evaluations. Performance of the PDM is very similar between the two evaluations. This is expected, as the PDM was not modified or altered between our initial evaluation on the ADED 2022 data and the subsequent blinded evaluation on the ADED 2023 data.

Table 1: PDM performance on non-blinded (ADED 2022) and blinded (ADED 2023) evaluations. "Slope of best fit line" is the slope of the line fit to the duration estimates and the true durations, with values less than 1 indicating underestimation. "R^2 of best fit line" is the R^2 value of the same best fit line. "Percent of estimates within a factor of 2x error" is the percent of duration estimates that are within a factor of 2x error from the true duration.

	**Non-blinded: ADED 2022**			**Blinded: ADED 2023**		
**Number of sensors**	**Slope of best fit line**	**R^2 of best fit line**	**Percent of estimates within 2x error**	**Slope of best fit line**	**R^2 of best fit line**	**Percent of estimates within 2x error**
**10**	-	-	-	0.95	0.80	86.8
**9**	-	-	-	0.94	0.82	85.5
**8**	0.89	0.84	89.3	0.98	0.82	82.9
**7**	0.89	0.83	86.4	0.99	0.82	81.6
**6**	0.88	0.81	84.6	1.02	0.84	78.9
**5**	0.88	0.82	81.6	1.17	0.88	77.0
**4**	0.87	0.81	77.0	1.12	0.77	69.4
**3**	0.76	0.70	66.7	1.23	0.77	63.9
**2**	0.70	0.63	59.3	1.07	0.54	65.5
**1**	1.20	0.62	63.3	1.03	0.37	61.8

S10 Effect of sensor placement optimization on duration estimates

Figure 1 in the main text shows how the performance of the PDM and the naive method change as the number of sensors installed on the METEC site decrease. For the subset of n sensors, Figure 1 shows the PDM and naive method results using the best possible arrangement of those n sensors. We selected the best sensor arrangement as follows. First, we created many different emission scenarios that were equally likely to occur on the METEC site. This was done by sampling many times from historical wind data on the site, and using these samples to simulate methane concentrations at all potential sensor locations from the different emission sources. Simulations were performed using the Gaussian puff atmospheric dispersion model. With a set of simulated concentrations for each emission scenario, we then tested the detection efficiency of all possible n-sensor subsets of the 10 sensor locations shown in Figure S5. We say that a given sensor configuration can detect a given emission scenario if at least 20% of the time steps during the emission had simulated concentration values over 0.5 ppm. The sensor configuration that successfully detected the most emission scenarios was selected as the optimal n-sensor arrangement for the METEC site.

In this section, we consider the performance of the PDM and naive method under sub-optimal sensor arrangements, as it is possible that sensor deployments in practice will not be fully optimized on each site. To do this, we employ the same optimization scheme described above, but instead of picking out the best n-sensor arrangement, we pick out the average n-sensor arrangement (that produces the detection efficiency closest to the average across all tested arrangements) and the worst n-sensor arrangement (that produces the worst detection efficiency across all tested arrangements).

Figures S9 through S11 show the PDM and naive method results on the ADED 2023 controlled releases under the best, average, and worst sensor arrangements. The same parity plot structure is used as in Figure 1 in the main

text, except that subfigure (a) now shows the duration estimates when using only 4 sensors instead of using all 10 sensors as in the main text. This was done because the best, average, and worst arrangements of a 10-sensor subset of 10 total sensors are all the same. Showing the 4-sensor subset in subfigure (a) highlights the differences in sensor arrangement optimization.

When using only 4 sensors, the best fit line for the PDM estimates remains relatively consistent: slope = 1.12 for the best arrangement, slope = 1.22 for the average arrangement, and slope = 1.12 for the worst arrangement. The best fit line for the naive method, however, drops quickly: slope = 0.63 for the best arrangement, slope = 0.57 for the average arrangement, and slope = 0.42 for the worst arrangement. This occurs because suboptimal sensor arrangements result in fewer detections by the CMS network, which increases the amount of CMS non-detect times that must be probabilistically addressed.

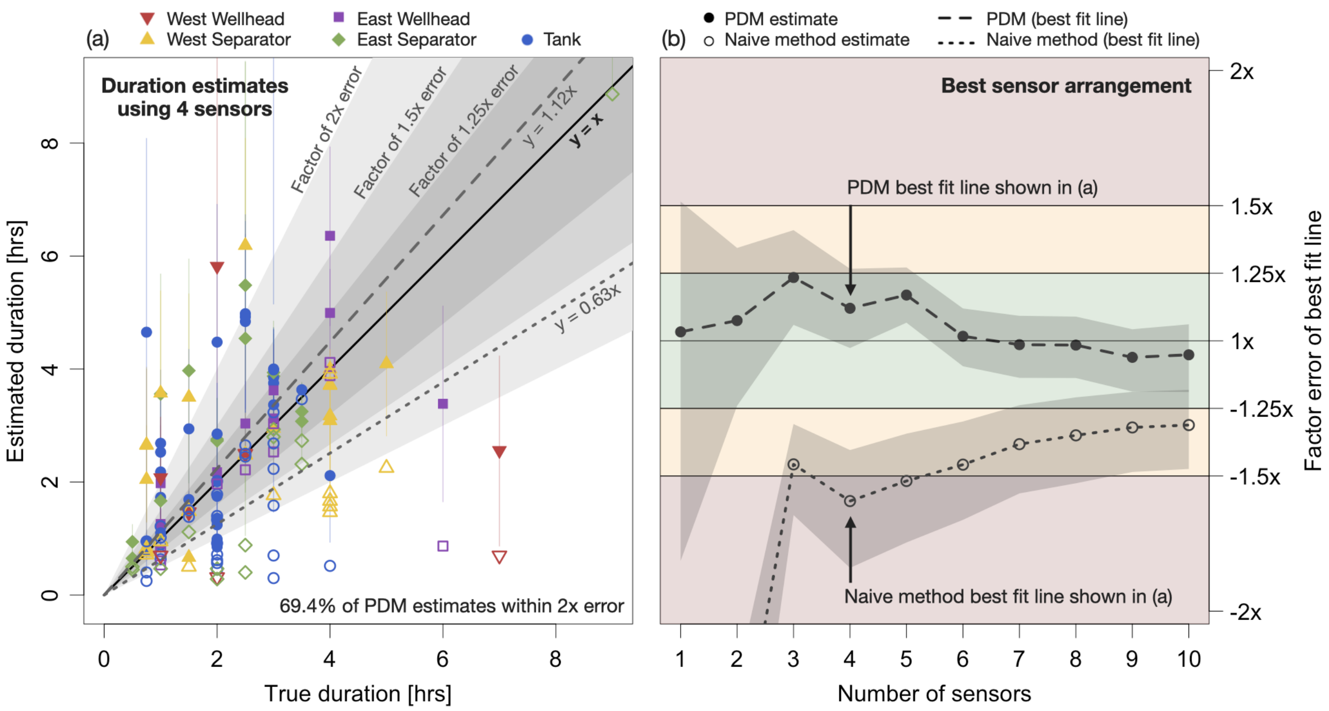

Figure S9: (a) Parity plot of estimated and true durations for the ADED 2023 controlled releases using the best 4-sensor arrangements. Solid and empty points correspond to duration estimates from the PDM and naive methods, respectively, with vertical lines showing the 90% interval from the PDM and color showing the true emission source. Dashed and dotted lines show the best linear fit to the PDM and naive estimates, respectively. Gray shaded regions show three different error regimes. (b) Factor of over or underestimation by the best linear fit to the PDM and naive estimates using different numbers of sensors and the best sensor arrangements. Gray shaded regions show the 95% confidence interval on the estimated slope. Negative factor differences indicate underestimation. Colored sections correspond to the three error regimes in (a). Note that the vertical scale is limited to [-2x, 2x] for visual clarity.

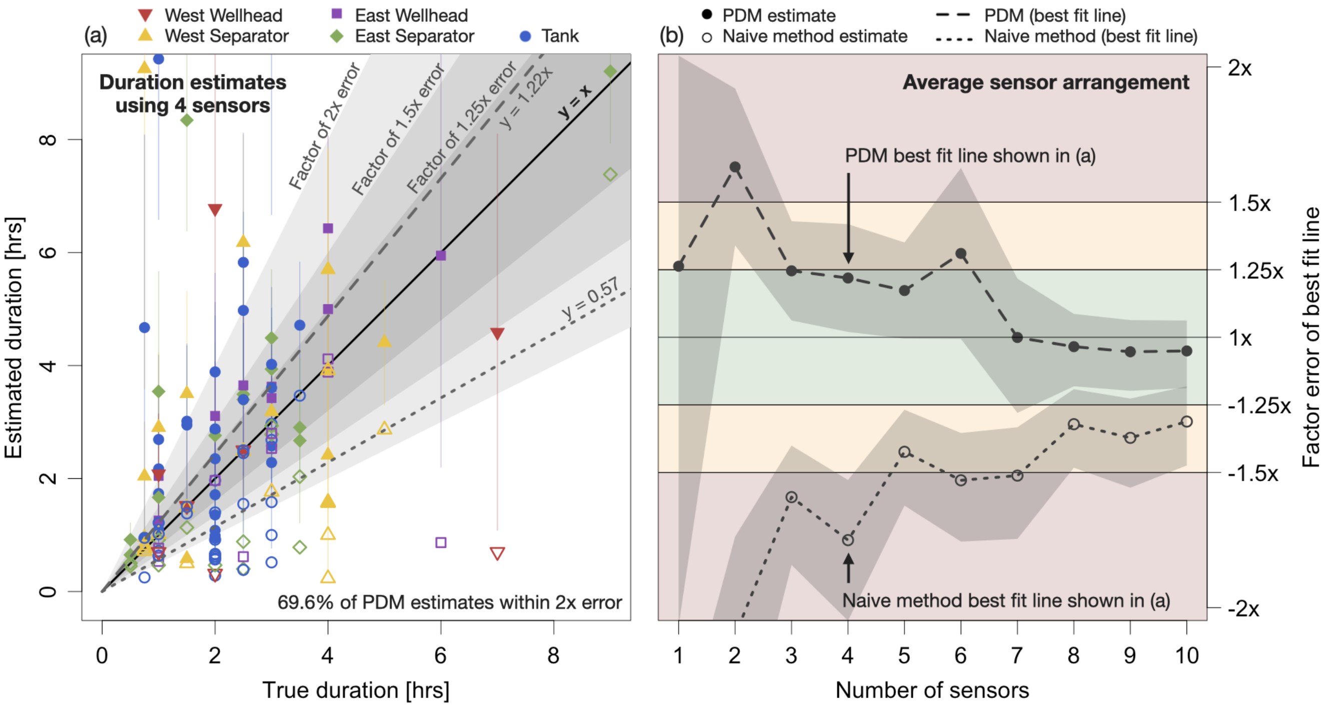

Figure S10: (a) Parity plot of estimated and true durations for the ADED 2023 controlled releases using the average 4-sensor arrangements. Solid and empty points correspond to duration estimates from the PDM and naive methods, respectively, with vertical lines showing the 90% interval from the PDM and color showing the true emission source. Dashed and dotted lines show the best linear fit to the PDM and naive estimates, respectively. Gray shaded regions show three different error regimes. (b) Factor of over or underestimation by the best linear fit to the PDM and naive estimates using different numbers of sensors and the average sensor arrangements. Gray shaded regions show the 95% confidence interval on the estimated slope. Negative factor differences indicate underestimation. Colored sections correspond to the three error regimes in (a). Note that the vertical scale is limited to [-2x, 2x] for visual clarity.

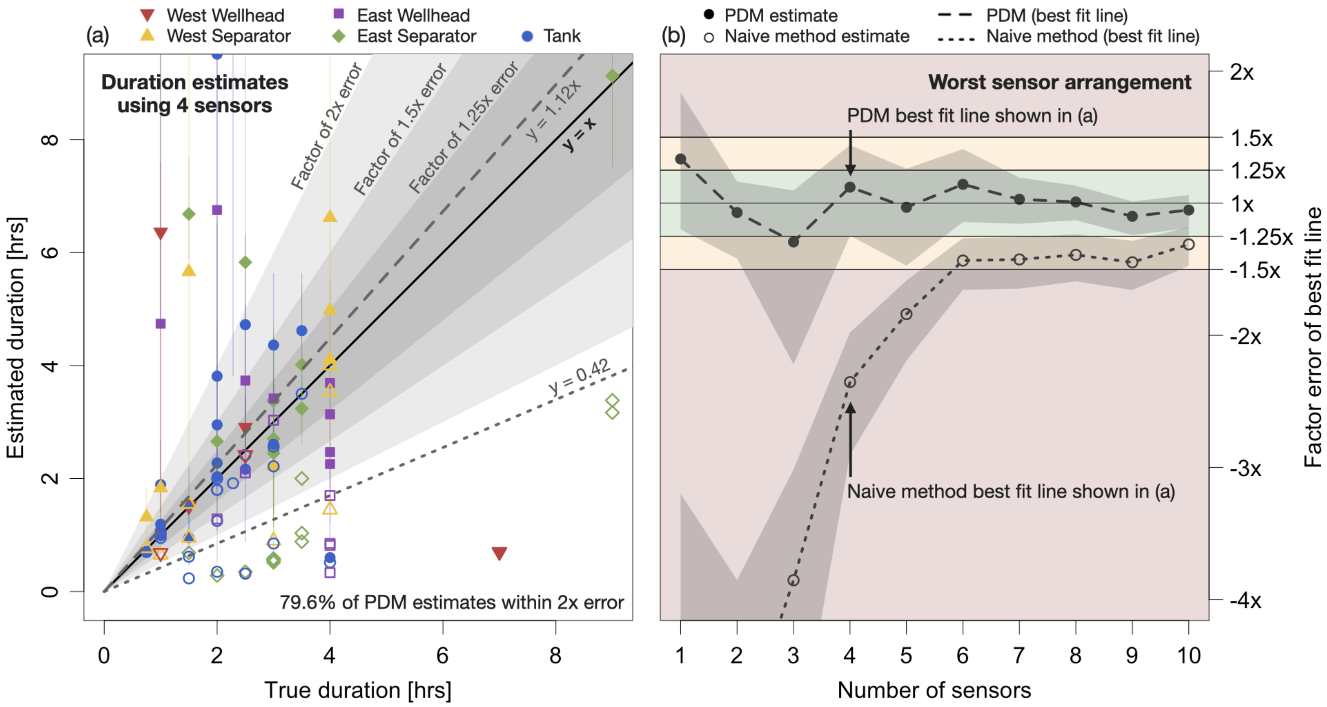

Figure S11: (a) Parity plot of estimated and true durations for the ADED 2023 controlled releases using the worst 4-sensor arrangements. Solid and empty points correspond to duration estimates from the PDM and naive methods, respectively, with vertical lines showing the 90% interval from the PDM and color showing the true emission source. Dashed and dotted lines show the best linear fit to the PDM and naive estimates, respectively. Gray shaded regions show three different error regimes. (b) Factor of over or underestimation by the best linear fit to the PDM and naive estimates using different numbers of sensors and the worst sensor arrangements. Gray shaded regions show the 95% confidence interval on the estimated slope. Negative factor differences indicate underestimation. Colored sections correspond to the three error regimes in (a). Note that the vertical scale is limited to [-4x, 2x] for visual clarity.

S11 Full detection, localization, and quantification results for AMI case study

Figures S12 and S13 show the full time series of detection, localization, and quantification results from the framework presented in [1] on the production site used as a case study in the main text. See Figure 2 in the main text for a schematic of this site. The gray line shows the minute-by-minute maximum of the background-removed concentration time series from all CMS sensors installed on the site (left vertical axis). Colored rectangles indicate naive events, with color representing the source estimate. Black points and vertical lines represent the estimated emission rates and 90% bootstrapped confidence intervals (right vertical axis).

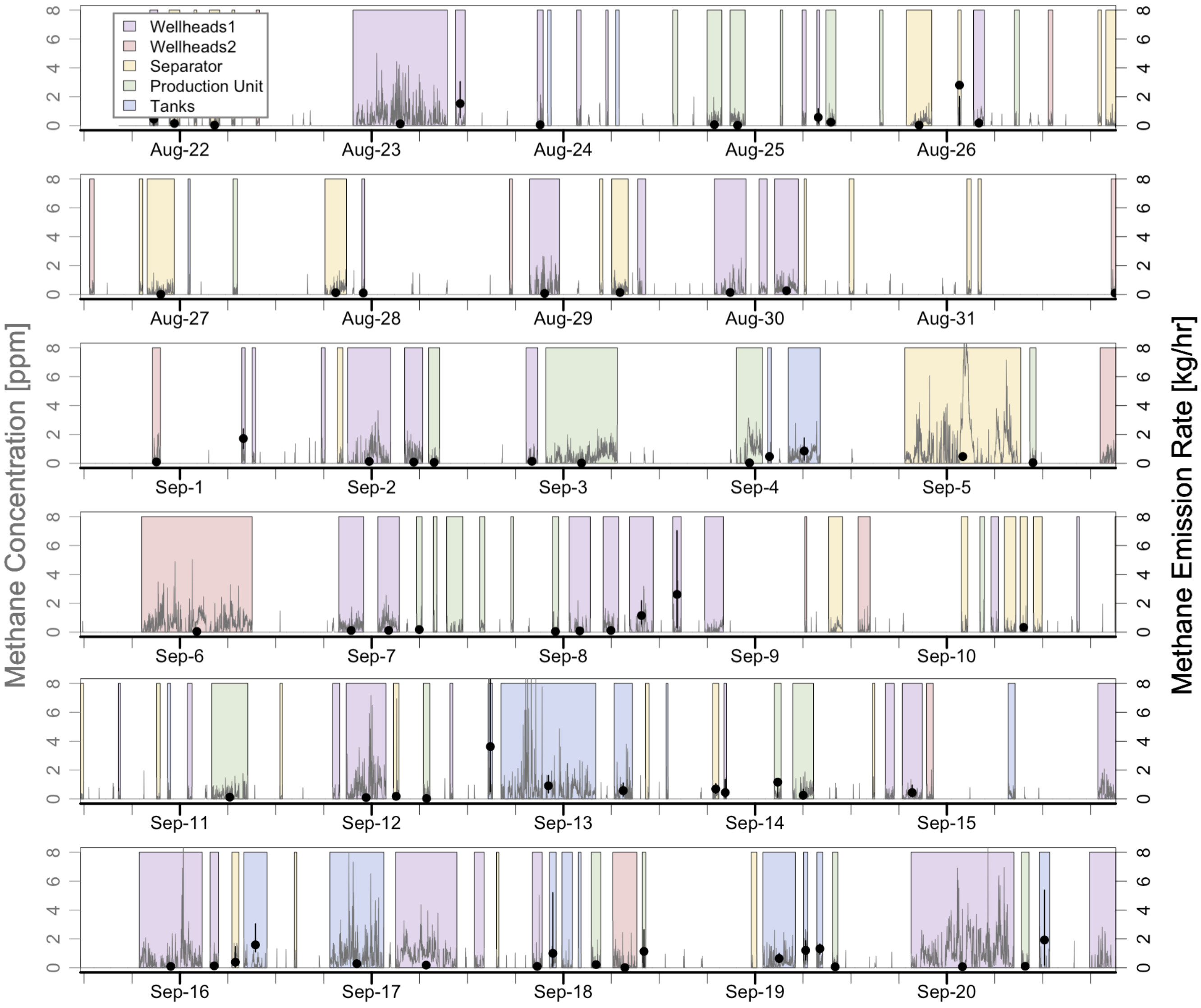

Figure S12: Time series of detection, localization, and quantification results on the site used as a case study in the main text. Time range spans August through September, 2023.

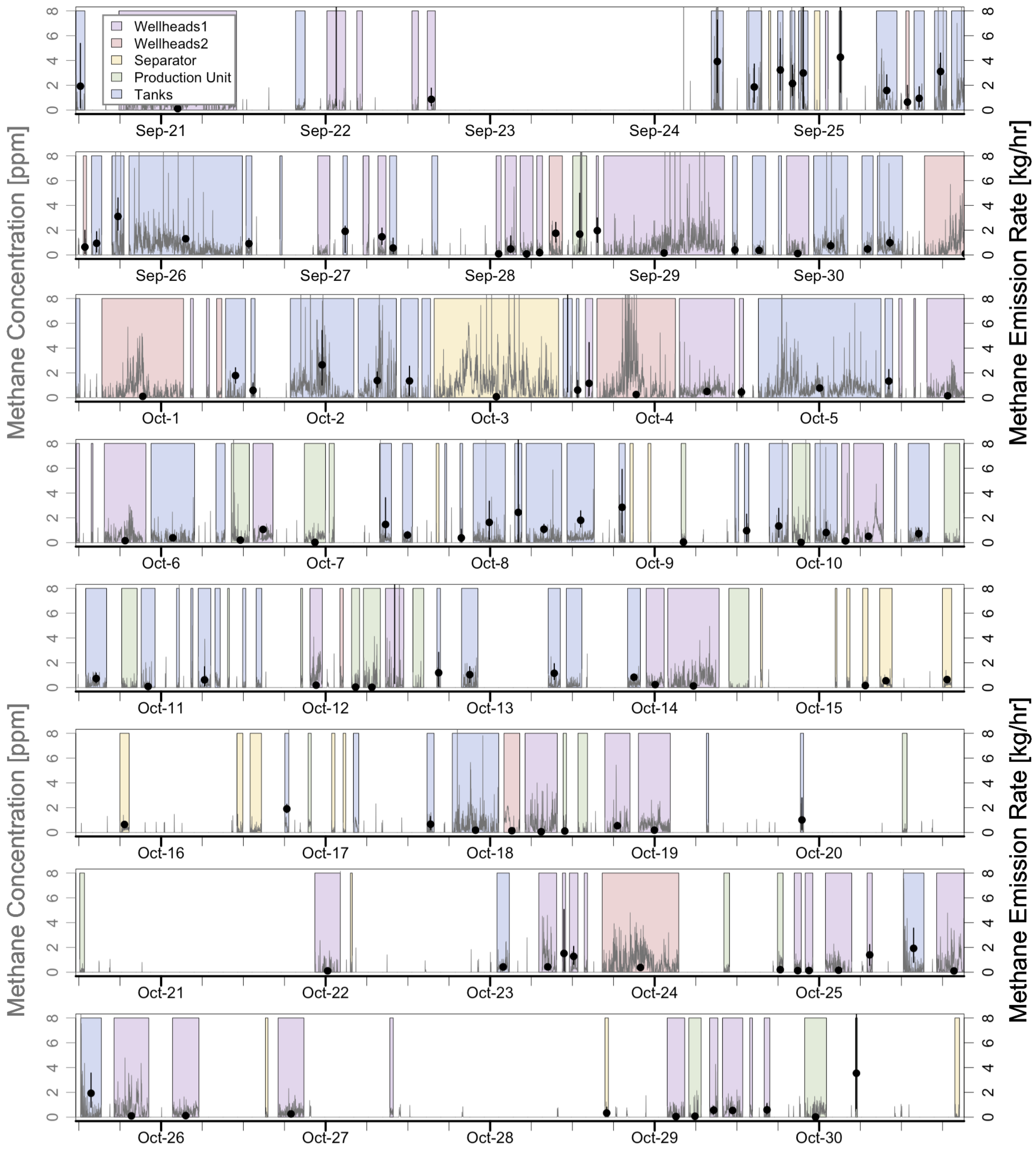

Figure S13: Time series of detection, localization, and quantification results on the site used as a case study in the main text. Time range spans September through October, 2023.

S12 Additional examples of bounding emission duration

In the main text, we demonstrate how the PDM can be used to bound the duration of a snapshot measurement. Figures S14 and S15 show two additional examples. For the example shown in Figure S14, the naive duration estimate (4.0 hrs) underestimates the mean (14.4 hrs) and maximum (20.5 hrs) duration estimates from the proposed method by a factor of 3.6x and 5.2x, respectively. For the example shown in Figure S15, the naive duration estimate (0.9 hrs) underestimates the mean (6.6 hrs) and maximum (11.4 hrs) duration estimates from the proposed method by a factor of 7.6x and 13.2x, respectively.

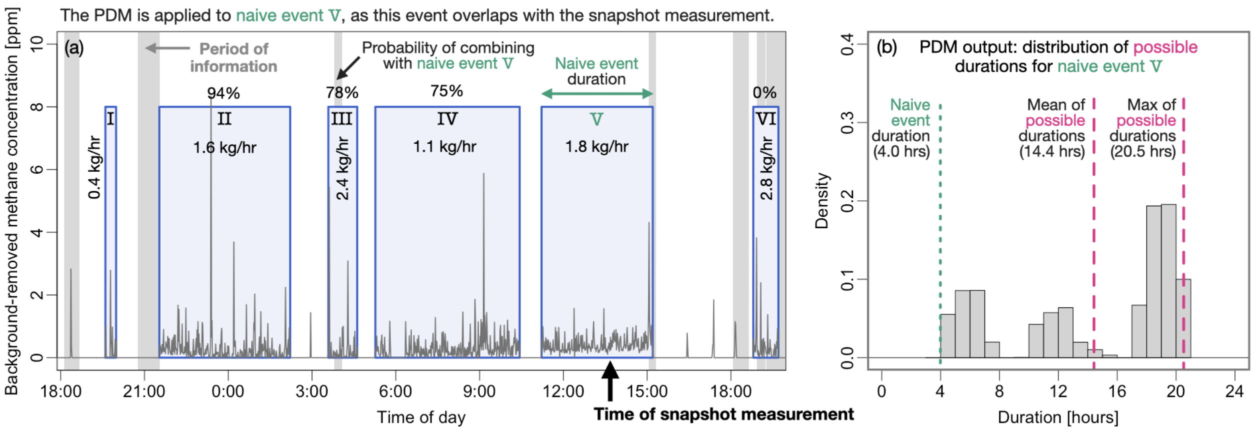

Figure S14: (a) Example snapshot measurement (time indicated by black arrow) and the overlapping CMS concentration data (spanning October 7, 2023 at 6:00pm to October 8, 2023 at 7:00pm). Enumerated boxes show the naive events, with color indicating the source estimate (color corresponds to the schematic in Figure 2(a)). Gray shaded regions mark periods of information. Percents indicate the probability of combining each event with the naive event that overlaps the snapshot measurement. (b) Distribution of possible durations from the PDM for naive event V and hence the overlapping snapshot measurement.

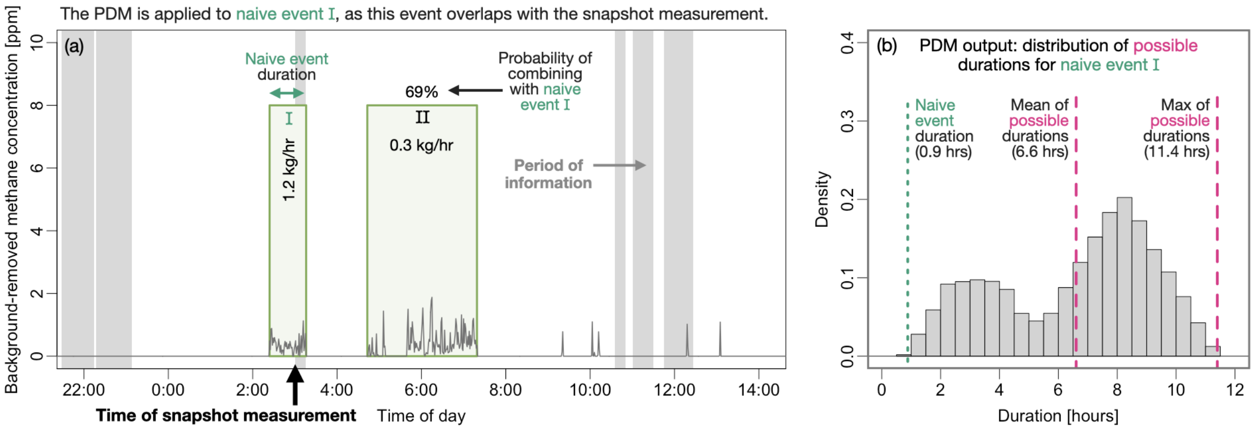

Figure S15: (a) Example snapshot measurement (time indicated by black arrow) and the overlapping CMS concentration data (spanning September 13, 2023 at 10:00pm to September 14, 2023 at 2:00pm). Enumerated boxes show the naive events, with color indicating the source estimate (color corresponds to the schematic in Figure 2(a)). Gray shaded regions mark periods of information. Percents indicate the probability of combining each event with the naive event that overlaps the snapshot measurement. (b) Distribution of possible durations from the PDM for naive event I and hence the overlapping snapshot measurement.

S13 Exhaustive search through all possible snapshot measurement times

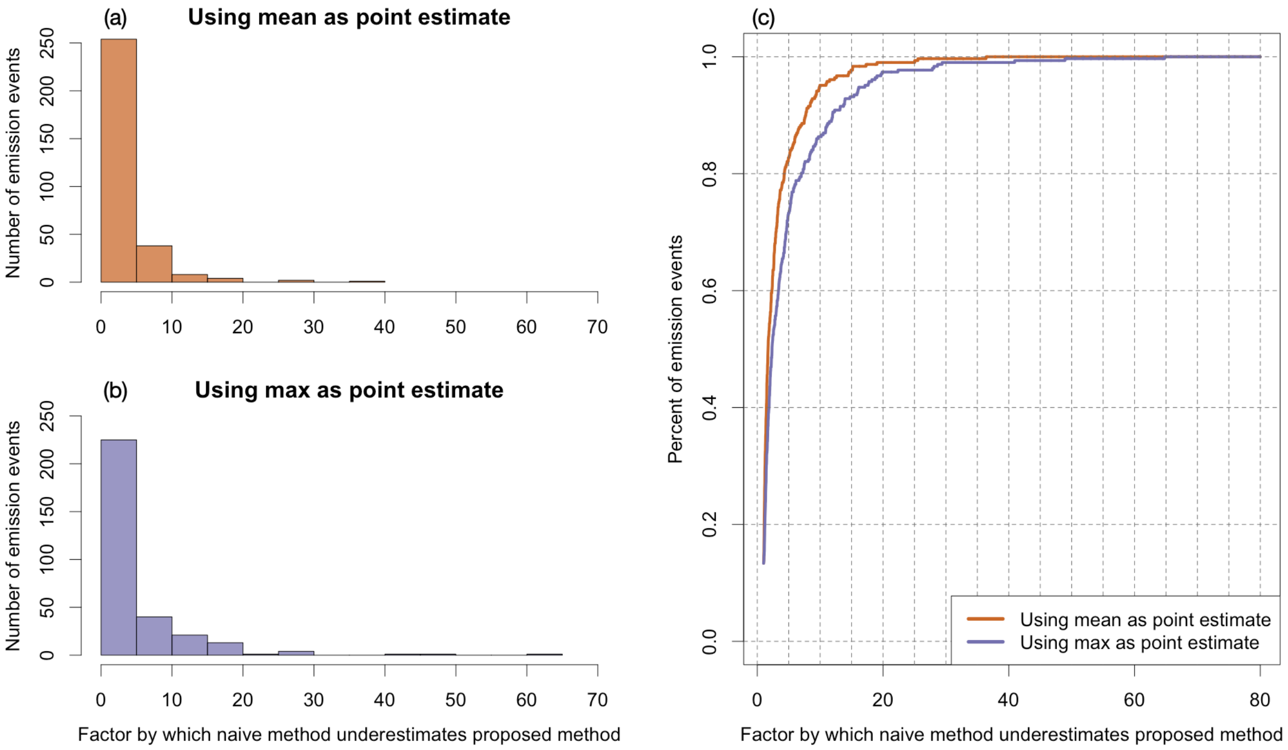

Figure S16: Result of exhaustive search through all possible snapshot measurement times on the oil and gas site used as case study in the main text. (a)-(b) Distribution of underestimation by the naive method, expressed as a factor, compared to the mean and maximum of the distribution of possible durations from the proposed method. (c) Empirical cumulative distribution function of the two distributions shown in (a) and (b).

In the main text and in Section S12 of the SI, we give a number of examples of using the PDM to bound the duration of snapshot measurements. As part of this analysis, we quantify the degree of underestimation from the naive method compared to the PDM.

However, these three examples represent only three possible times in which a snapshot measurement may have occurred. To more fully probe the extent of possible underestimation by the naive method, we consider all possible snapshot measurement times. For each snapshot measurement time, we compare the duration of the overlapping naive event to the mean and maximum of the distribution of possible durations from the PDM.

Figure S16 shows the results of this exhaustive search through all possible snapshot measurement times. Figures S16(a) and S16(b) show the distribution of underestimation by the naive method, expressed as a factor, compared to the mean and maximum of the distribution of possible durations from the PDM, respectively. For example, a factor of 2x means the duration estimate from the PDM is twice as long as the duration estimate from the naive method.

Figure S16(c) shows the empirical cumulative distribution function of the two distributions to better highlight the differences between using the mean and the maximum of the PDM as a point estimate of duration.

S14 Comparison of methods for estimating emission durations

Table 2: Strengths and weaknesses of various duration estimation methods.

	**Strengths**	**Weaknesses**
**CMS using PDM**	- Near real time measurements provide granular duration estimates.<br>- CMS can detect small emissions, which will be useful if the EPA reporting threshold is lowered in the future.<br>- The PDM addresses a key limitation of CMS: non-detect times when wind blows emitted methane between the sensors.	- Errors in location estimate can propagate to duration estimates, resulting in events not being combined.<br>- Quantification of CMS non-detect times is subject to errors in the dispersion model.<br>- Assumes a single emission source, which is unrealistic on complex sites with many possible emission sources.
**CMS using naive method**	- No strengths over CMS using the PDM.	- Does not account for CMS non-detect times, which can lead to large underestimation of emission durations.
**Aerial survey-based technologies**	- Does not require installation of equipment on each site, and therefore can measure many sites relatively easily.<br>- Provides good coverage of the site, which minimizes errors in the duration estimates resulting from errors in the localization estimates	- Often long gaps between subsequent measurements of a given source, meaning that duration estimates are often a conservative upper bound at the individual source-level.
**Satellites**	- Potential for relatively high frequency measurements of individual sources (i.e., daily). However, the measurement frequency is often less in practice due to suboptimal retrieval conditions.	- Can only detect very large emissions, and therefore, can only bound the duration of these very large emissions.<br>- Detection limits are still larger than the 100 kg/hr EPA reporting threshold.
**SCADA systems**	- High frequency data, at the second- or minute-level<br>- Many oil and gas operators already have these systems installed on their sites.	- Duration estimates are not based on actual measurements of methane, but rather on other variables such as tank pressures and temperatures. It is not yet clear if methane emissions can always be seen in these data.

S15 PDM limitations and next steps

It is important to emphasize the limitations of the PDM as currently implemented. First, the PDM assumes a single emission source for all detected emission events. This limits the accuracy of the PDM on complex sites where the single source assumption breaks down, as errors in the source location estimates will impact the accuracy of the information mask. We are currently developing a Bayesian model for localizing multi-source emissions, which will significantly improve the accuracy of the PDM on complex sites. Second, the PDM assigns zero probability of combining adjacent naive events if their source estimates are different, which again means that localization errors can propagate to errors in the duration estimates. Addressing this limitation will require methods for quantifying uncertainty in the localization estimates, which are currently under development. Finally, the information mask is subject to errors in the Gaussian puff dispersion model, which can result in naive events that occur during periods of no information (e.g., naive events I, III, and IV in Figure 3 in the main text). This could be improved by running the Gaussian puff model in a stochastic manner by perturbing the wind data and dispersion parameters to provide a measure of uncertainty in the resulting information mask.

References

[1] W. S. Daniels, M. Jia, and D. M. Hammerling, "Detection, localization, and quantification of single-source methane emissions on oil and gas production sites using point-in-space continuous monitoring systems," *Elementa: Science of the Anthropocene*, vol. 12, p. 00110, Mar. 2024. https://doi.org/10.1525/elementa.2023.00110.

[2] M. Jia, W. S. Daniels, and D. M. Hammerling, "Comparison of the Gaussian plume and puff atmospheric dispersion models for methane modeling on oil and gas sites," *ChemRxiv*, May 2023. https://doi.org/10.26434/chemrxiv-2023-hc95q-v2 (last accessed October 15, 2024).

[3] F. Pasquill, "The estimation of the dispersion of windborne material," *Meteorological Magazine*, vol. 90, pp. 33–49, 1961.

[4] B. D. Turner, "Workbook of atmospheric dispersion estimates," Tech. Rep. 742-R-70-001, U.S. Environmental Protection Agency, 1970.

[5] EPA, "Workbook for plume visual impact screening and analysis (revised)," Tech. Rep. 450-4-88-015, U.S. Environmental Protection Agency, 1992.

[6] Project Canary. https://www.projectcanary.com/ (last accessed October 15, 2024).

[7] R.M. Young Company, "ResponseONE Ultrasonic Anemometer Model 91000," Tech. Rep. 91000-90(F), 2021. https://www.youngusa.com/wp-content/uploads/2016/12/91000-90F.pdf (last accessed October 15, 2024).

[8] QUBE Technologies. https://www.qubeiot.com/ (last accessed October 15, 2024).

[9] Z. Chen, S. H. El Abbadi, E. D. Sherwin, P. M. Burdeau, J. S. Rutherford, Y. Chen, Z. Zhang, and A. R. Brandt, "Comparing Continuous Methane Monitoring Technologies for High-Volume Emissions: A Single-Blind Controlled Release Study," *ACS ES&T Air*, June 2024. https://doi.org/10.1021/acsestair.4c00015.
